# Nickel–Molybdenum Nanoparticles Anchored on Molybdenum Oxide as High-Performance Electrocatalyst for Hydrogen Production in Alkaline Water Electrolysis

**DOI:** 10.1021/acscatal.5c05933

**Published:** 2026-02-15

**Authors:** Anna K. Müller, Stefan Loos, Christian I. Bernäcker, Aaron Naden, Felix Heubner, Thomas Weißgärber

**Affiliations:** † 130437 Fraunhofer Institute for Manufacturing Technology and Advanced Materials IFAM, Winterbergstraße 28, 01277 Dresden, Germany; ‡ School of Chemistry, 7486 University of St Andrews, North Haugh, St Andrews KY16 9ST, U.K.; § Faculty Mechanical Engineering, Institute of Material Science, Chair Powder Metallurgy, TUD Dresden University of Technology, 01062 Dresden, Germany

**Keywords:** water electrolysis, hydrogen evolution reaction, electrocatalyst, NiMo, MoO_2_

## Abstract

The development of efficient and cost-effective electrocatalysts for alkaline water electrolysis is crucial for advancing hydrogen production technologies. Herein, we report the synthesis and characterization of a highly active, platinum-group-metal-free electrocatalyst for the hydrogen evolution reaction in alkaline media. The catalyst is obtained via the exsolution of Ni–Mo alloy nanoparticles anchored on MoO_2_ during the reduction of a NiMoO_4_ precursor grown on porous nickel substrates. It demonstrates high catalytic activity, achieving benchmark performance with an overpotential of −89 mV and a cell voltage of 1.71 V at a current density of 1 A/cm^2^. Electrochemical testing, including operation at high current densities under industrially relevant conditions (80 °C, 30 wt % KOH, flowing electrolyte), validates its suitability for large-scale applications. Importantly, structural analysis reveals the presence of Ni-rich Ni_10_Mo particles on MoO_2_ rather than the often-proposed Ni_4_Mo alloy, providing insight into the actual active phase of Ni–Mo catalysts. This distinction advances the fundamental understanding of Ni–Mo chemistry and offers guidance for the rational design of high-performance, platinum-group-metal-free catalysts for hydrogen evolution.

## Graphical abstract

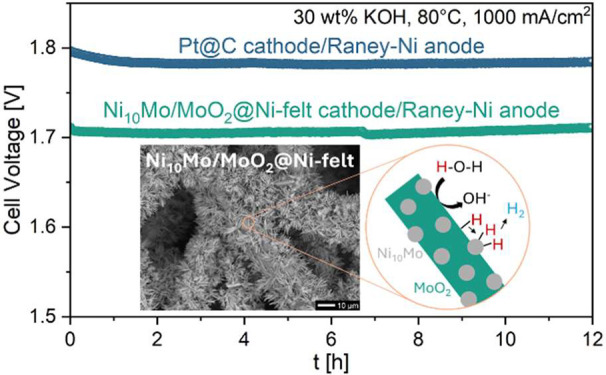

## 1 Introduction

The climate change is one of the biggest challenges of our time. Between 2011 and 2020, the global surface temperature was 1.1 °C higher than the preindustrial level (1850–1900).^1^ The aim of limiting the global warming to 1.5 °C is only achievable if the emission of greenhouse gases is reduced drastically in the near future.^1^ Hydrogen produced using renewable energy provides a clean energy source that can substitute fossil fuels and reduce carbon dioxide emissions. Water electrolysis is a key technology to produce green hydrogen, but it requires high efficiency and sufficiently high current densities to be economically viable.

Water splitting needs an energy input of 237 kJ/mol under standard conditions, which leads to a reversible cell voltage of 1.23 V.^2^ A higher voltage must be applied to overcome the electrical and other transport resistances and kinetic activation barriers which can be tackled by highly active electrocatalysts.

The electrical resistance in an electrolysis cell arises from the electrical conductivity of the electrodes, the concentration of the bulk electrolyte, the ionic conductivity inside the separator, as well as gas bubble effects. The activation overvoltage depends on the electrode material.^3^ With the use of electrocatalysts this overpotential at the anode and cathode can be reduced and the efficiency of hydrogen production via electrolysis can be improved. Highly active catalysts reduce the energy demand and costs of hydrogen production and, therefore, enable the widespread use of this technology.

The most relevant processes for water electrolysis are proton exchange membrane (PEM) electrolysis, solid oxide electrolyzer cell (SOEC) and alkaline water electrolysis (AWE), including anion exchange membrane (AEM) technology. Of these technologies, the AWE has the advantages that it allows the use of low cost electrocatalysts instead of noble metal catalysts used in PEM and operates at significantly lower temperatures (65–100 °C) than SOEC (>800 °C).^2,4^

In alkaline media the hydrogen evolution reaction (HER) involves two steps. The first step is the Volmer reaction 1 in which water dissociates and a hydroxide ion is released while hydrogen is adsorbed and accepts one electron.

$$H_{2}O+e^{-}\to H_{\mathrm{ad}}+{\mathrm{OH}}^{-}$$ 1

The second step is either the Tafel step ([Disp-formula eq2]), in which two adsorbed hydrogen atoms combine to form hydrogen gas

$$H_{\mathrm{ad}}+H_{\mathrm{ad}}\to H_{2}$$ 2

or the Heyrovsky step ([Disp-formula eq3]), in which adsorbed hydrogen reacts with water to hydrogen gas and one hydroxide ion.

$$H_{\mathrm{ad}}+H_{2}O+e^{-}\to H_{2}+{\mathrm{OH}}^{-}$$ 3

Platinum is a benchmark catalyst to accelerate the sluggish kinetics of the hydrogen evolution reaction (HER) in alkaline media. However, due to its high cost and scarcity it needs to be replaced with earth abundant and low-cost alternatives that show similar activity and comparable stability in alkaline media. As an earth abundant and cost-effective metal with good HER activity, high electrical conductivity and corrosion resistance in alkaline environment nickel (Ni) is a promising candidate. It has been shown that alloying Ni with other transition metals (TM) improves the HER performance. The HER activity of Ni-TM-alloys can be ranked in the following order: Ni–Mo > Ni–Zn > Ni–Co > Ni–W > Ni–Fe > Ni–Cr > Ni plated Steel.^5^ In the literature, Ni- and Mo-based materials are frequently reported as promising non-noble catalysts with high activity toward the HER.^6–21^ The high HER activity of Ni–Mo-alloys can be explained by Brewer’s hypo-hyper-d-electronic theory. When Mo, a TM with a less than half-filled d orbital, is alloyed with Ni, a TM with nearly full d orbital, a strong electronic interaction occurs.^22^ The hypo-hyper-d-electronic combination leads to a strong bonding effectiveness and, therefore, very stable intermetallic phases.^23^ Furthermore, it results in a modulated increased electronic density of states, which has been associated with enhanced catalytic activity.^23,24^ Luo et al.^24^ describe that catalysts with heterostructures of Ni_*x*_Mo_*y*_ alloys and oxides achieve higher activity than the pure alloy or oxide. This is because the alloy and the oxide play complementary roles for the HER. MoO_*x*_ promotes the water dissociation during the Volmer step by breaking H–OH bonds. The Ni_*x*_Mo_*y*_ alloy has an optimized hydrogen adsorption energy, because Mo influences the electronic structure of Ni, leading to a downshift of the d-band center of Ni. This improves the bond strength between Ni and adsorbed hydrogen closer to the optimum, where adsorption is neither too strong nor too weak, as required by the Sabatier principle for optimal catalytic performance. This leads to an accelerated Heyrovsky or Tafel step.

Xiao et al.^25^ demonstrated impressive performance of a Ni–Mo cathode in combination with a Ni–Fe anode in an alkaline polymer electrolyte water electrolyzer operating with pure water. At a current density of 0.4 A/cm^2^ it exhibited a cell voltage of 1.80–1.85 V at 70 °C. The Ni–Mo HER catalyst was not further analyzed, so it is unknown what properties such as morphology, composition, crystal structure or electronic structure are responsible for the high activity.

In particular, the combination of Ni_4_Mo and MoO_2_ has been reported as a highly active HER catalyst by several research groups. Zhang et al.^12^ obtained Ni_4_Mo nanoparticles on MoO_2_ cuboids via hydrothermal reaction followed by a reduction. The catalyst exhibited an overpotential of −12 mV at a current density of −10 mA/cm^2^ and a Tafel slope of 30 mV/dec in 1 M KOH. An et al.^15^ constructed MoO_*x*_ nanosheets with Ni_4_Mo nanoparticles on a copper substrate via a one-step galvanization. The Ni_4_Mo/MoO_*x*_/Cu catalyst had an overpotential of −16 mV at −10 mA/cm^2^ and a Tafel slope of 64 mV/dec in 1 M KOH. Meng et al.^26^ reported a Ni_4_Mo/MoO_*x*_ catalyst similar to that of Zhang et al. which achieved –10 mA/cm^2^ at −10 mV. Tian et al.^27^ synthesized Ni_4_Mo nanostructures on MoO_2_ that showed an overpotential of −23 mV at −10 mA/cm^2^ and a Tafel slope of 47 mV/dec. In line with many others, they propose that the Ni–Mo-alloy composition in the catalyst is Ni_4_Mo, but it is difficult to determine the exact composition of the nanoparticles that are present only as a thin layer on a substrate. In order to establish rational synthetic strategies for catalyst preparation, the investigation of the composition and structure needs further evidence and might also depend on experimental details during the production process and the mechanism of MoO_2_/Ni_*x*_Mo_1–*x*_ formation.

In this study, a composite material comprising Ni–Mo alloy nanoparticles anchored on MoO_2_ cuboids is demonstrated to be a highly active HER catalyst for AWE under industrial conditions (80 °C, 30 wt % KOH, high current densities, flowing electrolyte). Structural analysis suggests the presence of nickel-rich Ni_10_Mo particles on MoO_2_ rather than the often-proposed Ni_4_Mo alloy composition.

## 2 Experimental Section

### 2.1 Chemicals and Materials

Nickel foam (Ni-foam) 450 μm pore size, 420 g/m^2^, thickness 1.6 mm was obtained from Alantum Europe GmbH (Munich, Germany), ammonium molybdate tetrahydrate ((NH_4_)_6_Mo_7_O_24_·4H_2_O) (99.0%) from Alfa Aesar, Thermo Fisher Scientific (Geel, Belgium), nickel nitrate hexahydrate (Ni­(NO_3_)_2_·6H_2_O) (99.0%) from Acros Organics, Thermo Fisher Scientific (Geel, Belgium), hydrochloric acid (HCl) from Avantor, delivered by VWR (Darmstadt, Germany) and potassium hydroxide (KOH) (Fe content ≤0.0005%) Sigma-Aldrich, Merck KGaA (Darmstadt, Germany). Nickel fiber felt 2Ni18–050 (Ni felt) 80% porosity, thickness 500 μm was purchased from NV Bekaert SA (Zwevegem, Belgium), Pt@C-fiber-felt from Freudenberg Performance Materials GmbH & Co. KG (Weinheim, Germany), Nickel foil (Ni foil) from Goodfellow GmbH (Hamburg, Germany) and Zirfon Perl UTP 220 and Zirfon Perl UTP 500 diaphragms from Agfa-Gevaert N.V. (Mortsel, Belgium).

### 2.2 Synthesis of Ni_10_Mo/MoO_2_-Coated Electrodes for Half-Cell Tests

The synthesis of the Ni_10_Mo/MoO_2_ electrocatalyst involved two steps. First, a hydrothermal reaction, in which NiMoO_4_·xH_2_O cuboids were grown on Ni foam. Two pieces of Ni foam (1.0 cm × 3.5 cm) were immersed in 4 M HCl, ethanol and deionized water successively to clean the foams and remove native surface oxides. 0.19 mmol (NH_4_)_6_Mo_7_O_24_·4H_2_O and 1.08 mmol Ni­(NO_3_)_2_·6H_2_O were dissolved in 35 mL deionized water and stirred for 30 min. The corresponding concentrations were 5.4 mmol/L (NH_4_)_6_Mo_7_O_24_·4H_2_O and 30.9 mmol/L Ni­(NO_3_)_2_·6H_2_O. The cleaned Ni foams were immersed into the solution in a glass lined autoclave. The autoclave was heated to 150 °C and kept at this temperature for 6 h before it was cooled down to room temperature. Then the foams were washed with deionized water and ethanol and dried at 60 °C for 1 h. The resulting precursor was denoted as NiMoO_4_@Ni-foam. In the second step, the precursor-coated foams were reduced at 500 °C for 0.5, 2, or 3 h in an Ar/H_2_ (95/5 vol %, 2 Nl/min) atmosphere. The resulting samples were designated as Ni_10_Mo/MoO_2_@Ni-foam-0.5h, Ni_10_Mo/MoO_2_Ni-foam-2h, and Ni_10_Mo/MoO_2_@Ni-foam-3h, respectively. The synthesis of the Ni_10_Mo/MoO_2_ coated substrates is depicted in Figure 1.

**Figure 1:**
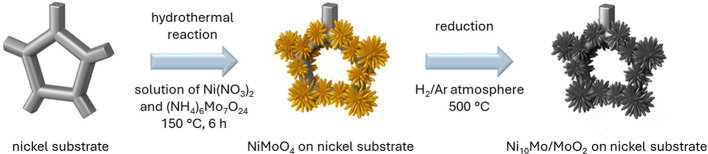
Schematic illustration of the synthesis of the Ni_10_Mo/MoO_2_ coated substrates.

### 2.3 Synthesis of Ni_10_Mo/MoO_2_-Coated Electrodes for Single-Cell Tests

The synthesis of the cathode material for single-cell tests was analogous to the synthesis for half-cell tests except for the used substrate and amount of metal salts. For cell tests the catalyst was deposited on larger substrates (3.0 cm × 3.3 cm) and on Ni foam as well as on Ni fiber felt. The resulting samples were designated as Ni_10_Mo/MoO_2_@Ni-foam and Ni_10_Mo/MoO_2_@Ni-felt, respectively. The amount of metal salts for the hydrothermal reaction was adjusted to the higher geometrical surface area. Specifically, 0.28 mmol (NH_4_)_6_Mo_7_O_24_·4H_2_O and 1.54 mmol Ni­(NO_3_)_2_·6H_2_O were dissolved in 35 mL deionized water. The corresponding concentrations were 8 mmol/L (NH_4_)_6_Mo_7_O_24_·4H_2_O and 44 mmol/L Ni­(NO_3_)_2_·6H_2_O. The reduction time was 2 h. The synthesis of the Ni_10_Mo/MoO_2_ coated substrates is depicted in Figure 1.

### 2.4 Synthesis of MoO_2_-Coated Electrodes for Half-Cell Tests

For the synthesis of the MoO_2_ coated electrode 1.80 mmol (NH_4_)_6_Mo_7_O_24_·4H_2_O were dissolved in 30 mL deionized water and stirred for 30 min. Two pieces of Ni foam (1 cm × 3.5 cm) were immersed in 4 M HCl, ethanol and deionized water successively to clean the foams and remove NiO. The cleaned Ni foams were immersed into the solution in a glass lined autoclave. The autoclave was heated to 160 °C for 4 h. Then the foams were washed with deionized water and ethanol and dried at 60 °C for 1 h. The precursor-coated foams were annealed at 500 °C for 2 h in Ar atmosphere. The resulting sample was denoted as MoO_2_@Ni-foam. The synthesis of MoO_2_@Ni-foam is depicted in Figure S1.

### 2.5 Material Analysis

Scanning electron microscopy (SEM), energy-dispersive X-ray spectroscopy and elemental mapping were carried out with a Jeol JSM-IT800 and a XFlash 6/30 EDX from Bruker. X-ray diffraction (XRD) patterns were recorded with a D8 Advance diffractometer from Bruker using monochromatic CuKα radiation. The XRD patterns were collected between 2 Theta = 20° and 100° with a step size of delta 2 Theta = 0.02° and with the counting time of 10 s per step. For the phase analysis using the Rietveld method, the computer program MAUD was used.^56^ The XRD measurements were employed mainly as a global method to exactly determine the lattice parameters of the phases and to quantify their crystallite sizes. Sequentially, the following parameters were refined for all phases: Background and scale parameters (background polynomial with 6 parameters, phase percentages), lattice parameter(s), temperature factor, crystallite size, microstrain. The arbitrary texture model was selected to respect the crystallite growth with preferred orientations. X-ray photoelectron spectroscopy (XPS) was conducted using a UHV system (SPECS, Berlin). A monochromatic X-ray source (XR50 with Aluminum, *E* = 1486.71 eV) equipped with a Focus 500 monochromator was used for excitation. The emitted photoelectrons were analyzed using a PHOIBOS 150 in fixed analyzer transmission (FAT) mode. Data evaluation was performed using CasaXPS (v2.3.18). Scanning transmission electron microscopy (STEM) measurements were performed on an FEI Titan Themis operated at 200 kV, equipped with a CEOS DCOR probe corrector and a SuperX energy dispersive X-ray spectrometer. High angle annular dark field (HAADF) STEM images were acquired with a probe convergence angle of 20 mrad and inner/outer collection angles of 91 and 200 mrad, respectively. The energy dispersive X-ray data were also extracted by line scans and mapping analysis to distinguish the composition of nanoparticles in different areas of the sample.

### 2.6 Electrochemical Measurements

The electrochemical tests were conducted with a Reference 3000 potentiostat from Gamry. All measurements were conducted in 30 wt % KOH at 80 °C. All key electrochemical measurements were independently repeated 2–3 times using separately prepared electrodes. The obtained results showed good reproducibility. For the electrochemical tests in a three-electrode setup, the catalyst-coated Ni foam was used as the working electrode (WE), a platinum wire as the counter electrode (CE) and a reversible hydrogen electrode (RHE) as the reference electrode. A two-compartment cell was employed, in which the compartments were separated by a Zirfon Perl UTP 500 diaphragm to prevent gas crossover. The working and reference electrodes were placed in one compartment, while the counter electrode was positioned in the other. ICP-OES analysis of the electrolyte before and after electrochemical testing and post-testing SEM-EDX analysis of the WE confirmed that no detectable platinum dissolution from the CE and redeposition on the WE occurred. Tafel plots were obtained from a series of galvanostatic measurements at defined current densities, each maintained for 60 s. For data evaluation, the average overpotential recorded during the last 30 s of each step was used. The galvanostatic measurements were performed at a current density of −1000 mA/cm^2^ for 5 h, with the potential recorded every 10 s.

All potentials in the half-cell measurements are referenced to the RHE and have been corrected for the ohmic drop caused by the electrolyte resistance. The uncompensated resistance (*R*_u_) was determined using the current interrupt method, in which the potential response is recorded immediately after interrupting the current. *R*_u_ is calculated from the instantaneous potential drop divided by the applied current.

The single-cell tests were performed in a zero-gap configuration with the Micro Flow Cell from ElectroCell. A schematic illustration is provided in Figure S2. The catalyst-coated substrate was used as the active layer at the cathode side. At the anode side an in-house made Raney-Ni mesh was used, following the synthesis procedure described in our previous work.^28^ On both sides a Ni foam (450 μm pore size) was used as the diffusion layer. The electrodes were separated by a Zirfon Perl UTP 220 diaphragm. The electrolyte flow was 55 mL/min at the anode and cathode. Tafel plots were obtained from a series of galvanostatic measurements at defined current densities, each maintained for 60 s. For data evaluation, the average overpotential recorded during the last 30 s of each step was used. The galvanostatic measurements were performed at a current density of 1000 mA/cm^2^ for 12 h or 670 mA/cm^2^ for 100 h, with the potential recorded every 30 s. The cell voltage was not corrected for the ohmic drop.

## 3 Results and Discussion

The synthesis of the Ni_10_Mo/MoO_2_ catalyst on Ni substrate is illustrated in Figure 1. It consisted of two steps. The first step was a hydrothermal reaction, where NiMoO_4_·xH_2_O was grown on the Ni substrate. Second, the precursor was turned into Ni_10_Mo/MoO_2_ via reduction at elevated temperature and under Ar/H_2_ 95/5 (v/v) atmosphere.

For XRD measurements, the precursor and catalyst (reduction for 2 h) were synthesized on Ni foil as substrate so the materials could be scratched off easily and characterized as powders to avoid predominant nickel-signals. Figure 2a shows the X-ray powder pattern of the precursor. NiMoO_4_ has two modifications that are stable under standard pressure. The α-phase is stable at low temperatures while the β-phase is stable at high temperatures. The conversion takes place at temperatures above 600 °C while heating and at temperatures below 250 °C while cooling.^29^ Both modifications crystallize in the monoclinic system, but they differ in the coordination sphere of the molybdenum ions. In the crystal structure of α-NiMoO_4_, molybdenum is octahedrally coordinated in [MoO_6_] units, whereas in β-NiMoO_4_, it is tetrahedrally coordinated in [MoO_4_] units.^29,30^ Hydrated NiMoO_4_·xH_2_O usually precipitates in poorly crystalline form, but via hydrothermal synthesis it can be obtained with high crystallinity.^31^ The diffraction pattern of the precursor is in good agreement with the pattern obtained by Eda et al.^31^ for NiMoO_4_·2.8 H_2_O. It has a triclinic crystal structure (space group *P*-1) with lattice constants *a* = 6.7791 Å, *b* = 6.8900 Å, *c* = 9.2486 Å, α = 76.681°, β = 83.960°, γ = 74.218°. The formation of NiMoO_4_ from the dissolved metal salts is shown in eq 4.

$${\mathrm{Ni}}^{2+}+{\mathrm{MoO}}_{4}^{2-}+xH_{2}O\to {\mathrm{NiMoO}}_{4}\cdot xH_{2}O$$ 4

**Figure 2:**
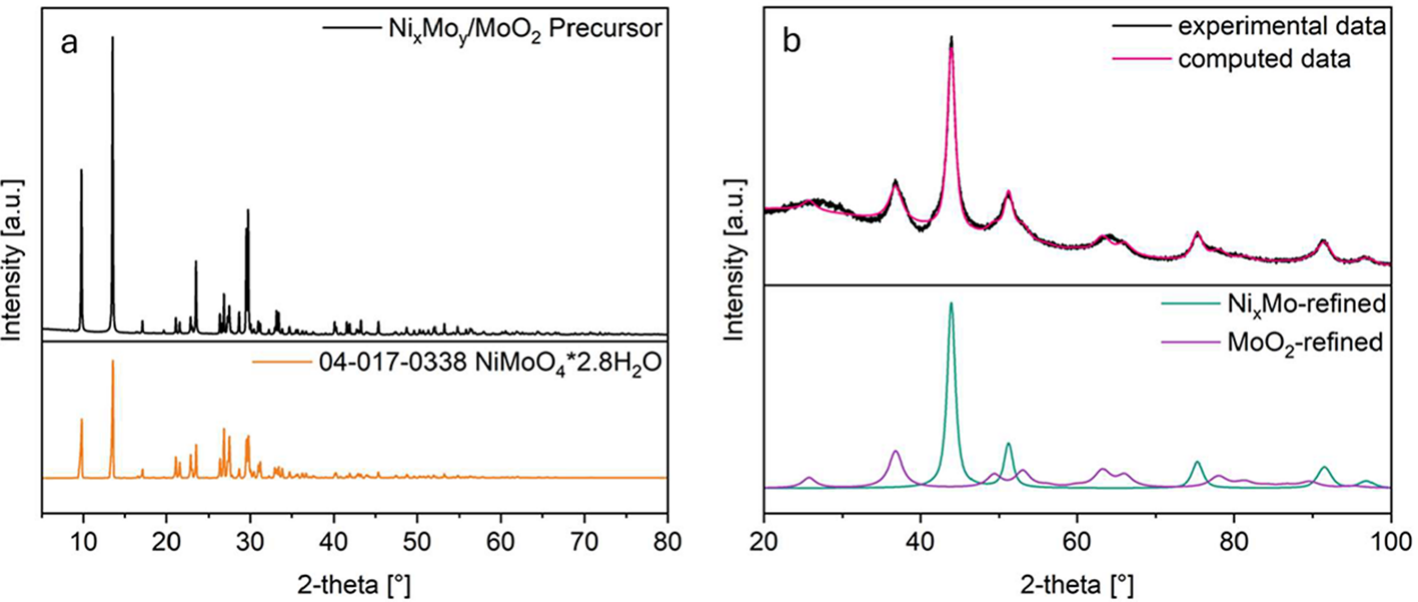
X-ray powder diffraction pattern of (a) NiMoO_4_ precursor and (b) Ni_10_Mo/MoO_2_ catalyst.

In the diffraction pattern of the Ni_10_Mo/MoO_2_ catalyst (Figure 2b) the peaks at 26, 37, 53, and 67° 2θ can be assigned to MoO_2_. The oxide crystallizes in monoclinic structure (space group *P*2_1_/*n*). The peaks at 44, 51, 75, 92, and 97° (2θ) match best with the peaks of the (111); (200); (220); (311), and (222) lattice planes of Ni_0,91_Mo_0,09_ found in the database ICDD PDF-4+ (2023). These peaks are the same as for pure Ni, but they are shifted to smaller angles (larger unit cells). This is due to the circumstance that up to 12.5 at% molybdenum can be substitutionally dissolved in the face-centered cubic (fcc) structure (space group *Fm*3̅*m*) of Ni,^32^ but the lattice parameters change as the atomic radius of Mo (1.45 Å) is bigger than the one of Ni (1.35 Å).^33^ The ratio of Ni:Mo was determined by refining the lattice parameter a to fit the diffraction pattern the best and using Vegard’s law. Therefore, every Ni–Mo-alloy with fcc structure from the database ICDD was plotted in a diagram that shows the lattice parameter a as a function of the Mo content in the alloy (Figure 3 and Table 1). The refined parameter *a* = 3.5657 Å corresponds to a Mo content of 9.2 at%. Accordingly, the chemical formula of the Ni–Mo-alloy is referred to as Ni_10_Mo. The formation of MoO_2_ and Ni_10_Mo is shown in eq 5. The diffraction patterns of Ni_10_Mo/MoO_2_-0.5h, Ni_10_Mo/MoO_2_-2h, and Ni_10_Mo/MoO_2_-3h are provided in Figure S3. These samples exhibit the same characteristic reflections of the Ni_10_Mo solid solution and the MoO_2_ phase, indicating that no additional crystalline phases are formed at shorter or longer reduction times. The diffraction pattern of MoO_2_@Ni-foam is provided in Figure S4.

$$10{\mathrm{NiMoO}}_{4}+22H_{2}\to 9\mathrm{Mo}O_{2}+{\mathrm{Ni}}_{10}\mathrm{Mo}+22H_{2}O$$ 5

**Figure 3:**
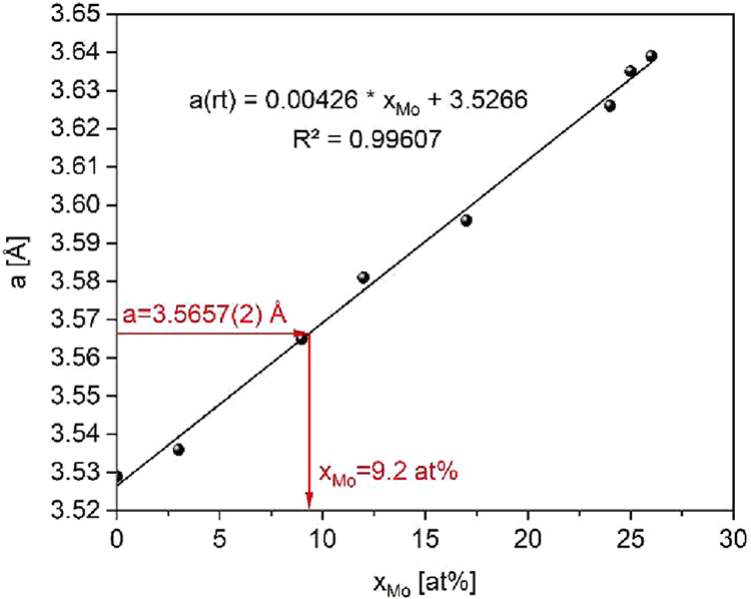
Lattice parameter a of Ni–Mo-alloys with fcc structure from the ICDD database in dependence of the molybdenum content.

**Table 1:** Lattice Parameter *a* of Ni–Mo-Alloys with the fcc Structure from the ICDD Database

formula	Mo content *x*_Mo_ [at %]	lattice parameter *a* [Å]	source
Ni	0	3.529	^34^
Mo_0.03_Ni_0.97_	3	3.536	^35^
Mo_0.09_Ni_0.91_	9	3.565	^36^
Mo_0.12_Ni_0.88_	12	3.581	^37^
Mo_0.17_Ni_0.83_	17	3.596	^38^
Mo_0.24_Ni_0.76_	24	3.626	^39^
Mo_0.25_Ni_0.75_	25	3.635	^40^
Mo_0.26_Ni_0.74_	26	3.639	^41^

The SEM images (Figure 4a–d) reveal that during the hydrothermal reaction the precursor compound NiMoO_4_·2.8H_2_O grows in the form of cuboids with a length of ∼10 μm and a width of 0.5–1.0 μm. These cuboids have a smooth surface and cover the Ni-foam densely. After reduction for 0.5 and 2 h (Figures S6a–d and 4e–h) the cuboid morphology is maintained, but their surface has become rough. Through exsolution, nanoparticles with a diameter of ∼10 nm formed on the surface. After a reduction for 2 h the nanoparticles cover the cuboids more densely than after 0.5 h. It is assumed that these nanoparticles consist of the Ni–Mo-alloy Ni_10_Mo and are socketed into MoO_2_ cuboids. MoO_2_ has metallic nature based on the Mo–Mo bonding in its distorted rutile structure and exhibits good electric conductivity.^42,43^ Furthermore, the cuboids offer a very high surface area for the metallic nanoparticles as highly active sites for the HER. After reduction for 3 h (Figure S6e–h), the morphology of the cuboids changed significantly, transforming into a highly porous, wrinkled structure with interconnected nanosheets. The nanosheets are densely covered with nanoparticles with a diameter of ∼100 nm. The SEM images of MoO_2_@Ni-foam are provided in Figure S5.

**Figure 4:**
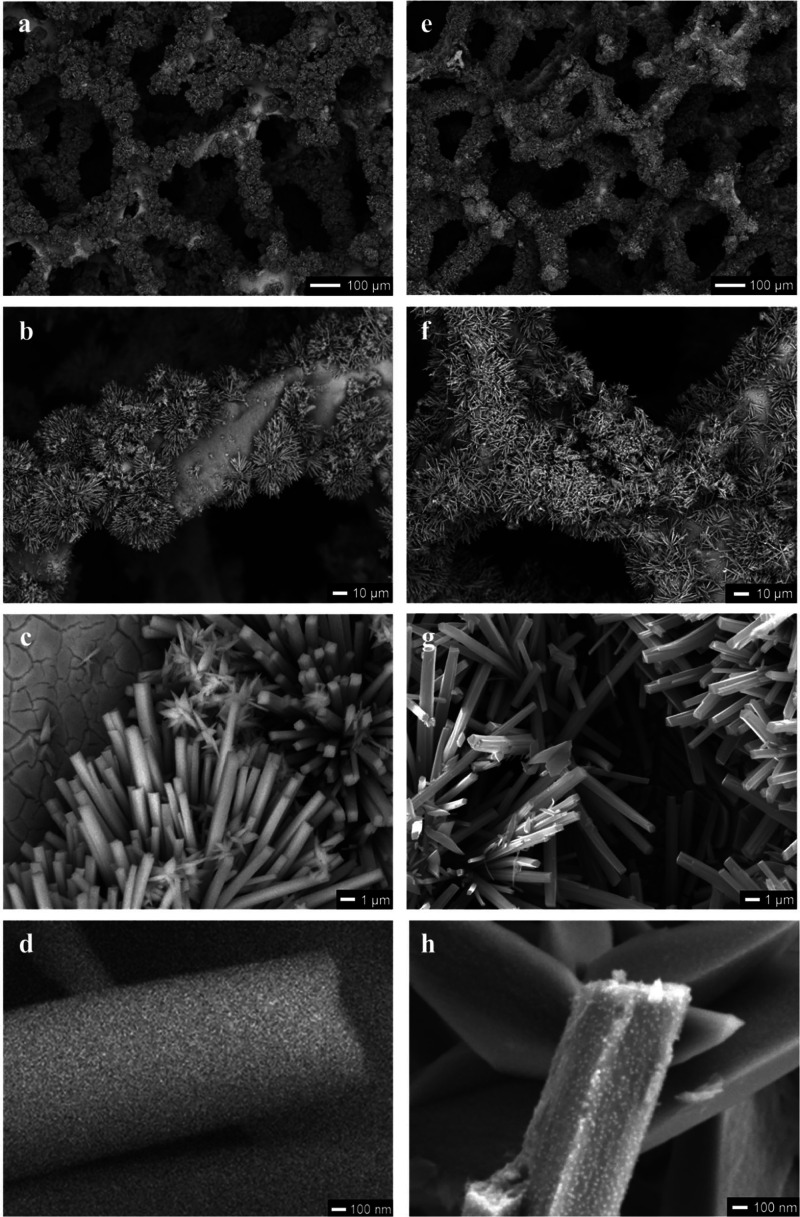
SEM images of NiMoO_4_@Ni-foam (a–d) and Ni_10_Mo/MoO_2_@Ni-foam-2h (e–h).

The TEM image of Ni_10_Mo/MoO_2_@Ni-foam-2h (Figure 5a) shows the top of a microcuboid structure decorated with nanosized particles like shown in Figure 4h. While the TEM contrast reveals the morphology, the corresponding EDS elemental mapping (Figure 5b) provides the compositional information. It shows that the cuboid body is Mo-rich, whereas the surface nanoparticles are enriched in Ni. This confirms that the catalyst consists of Ni-rich Ni–Mo alloy nanoparticles anchored on MoO_2_ cuboids. Individual TEM-EDS maps for Ni and Mo are shown in Figure S7. HR-TEM analysis was conducted to determine the composition of the Ni–Mo nanoparticles. The *d*-spacings of several (*hkl*) planes was measured at multiple spots of the sample and the corresponding lattice parameters and Mo contents in the solid solution were calculated. The results are summarized in Table S1. The average Mo content obtained from HR-TEM was 7.6 ± 8.2 at%, which is in reasonable agreement with the XRD-derived value of 9.2 at%. However, the individual measurements show large deviations. This reflects the significantly higher uncertainty in lattice parameter determination via HR-TEM. Despite these uncertainties, the HR-TEM results support the conclusion that the nanoparticles are Ni-rich and confirm that their composition is closer to Ni_10_Mo than Ni_4_Mo.

**Figure 5:**
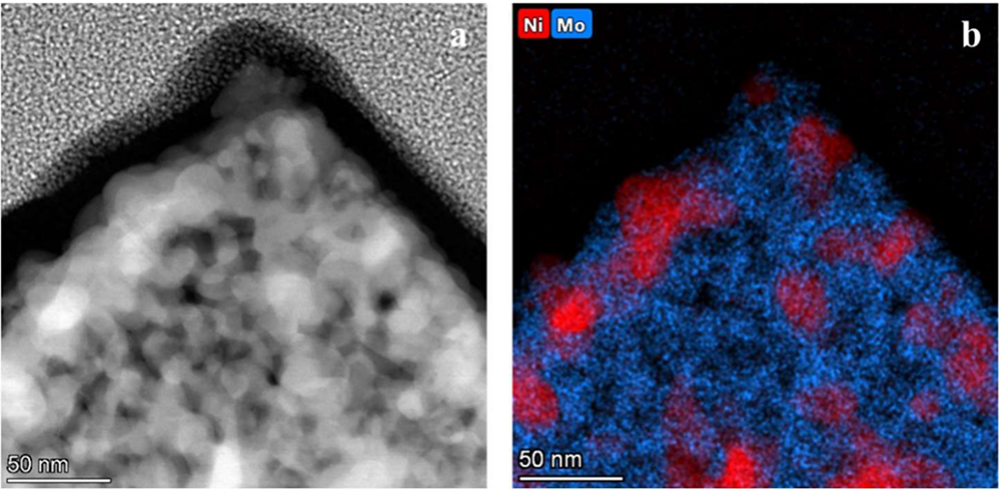
TEM image of a micro cuboid with nanoparticles (a) and TEM-EDS mapping of Ni and Mo (b).

XPS was conducted to analyze the chemical composition and surface oxidation states of the catalyst. The ratio of metallic Ni to metallic Mo especially was of interest to confirm the Ni_10_Mo composition of the nanoparticles that was determined from the XRD analysis. The Mo 3d spectrum (Figure S8a) reveals the presence of multiple oxidation states: Mo^0^, Mo^4+^, Mo^5+^, and Mo^6+^. The Ni 2p spectrum (Figure S8b) exhibits peaks than can be assigned to Ni^0^ and Ni^2+^. This observation deviates from expectations based on the diffraction pattern, which indicated only MoO_2_ and a Ni–Mo alloy. A possible explanation is that a fraction of the precursor was not fully reduced, leading to the formation of amorphous NiMoO_4_. However, it was not possible to reliably quantify the Ni^0^/Mo^0^ ratio, as the Mo^0^ signal was too weak to be fitted with sufficient accuracy. Consequently, the XPS data cannot contribute to the analysis of the composition. Additionally, one has to take into account that XPS does only deliver information about the outer surface (2–3 nm).

The electrocatalytic HER performance was tested with a three-electrode arrangement (3EA) in N_2_-saturated 30 wt % KOH at 80 °C. All potentials are referenced to the RHE and the potentials have been corrected for the ohmic drop caused by the electrolyte resistance. For comparison, a MoO_2_ coated Ni foam (MoO_2_@Ni-foam) was prepared and tested as well as a plain Ni-foam. Repetition measurements are provided in Figures S10–S12.

The galvanostatic measurement of Ni_10_Mo/MoO_2_@Ni-foam-2h, MoO_2_@Ni-foam and the pure Ni-foam at −1 A/cm^2^ is shown in Figure 6a. The electrochemical characterization of Ni_10_Mo/MoO_2_@NF-0.5h and Ni_10_Mo/MoO_2_@Ni-foam-3h is shown in Figure S9. After measuring at a current density of –1 A/cm^2^ for 5 h, the catalyst reduced for 2 h Ni_10_Mo/MoO_2_@Ni-foam-2h showed the lowest overpotential of only −89 mV. The performance of Ni_10_Mo/MoO_2_@Ni-foam-2h was much better than the one of MoO_2_@Ni-foam (−239 mV), Ni-foam (−446 mV), Ni_10_Mo/MoO_2_@Ni-foam-0.5h (−404 mV) and Ni_10_Mo/MoO_2_@Ni-foam-3h (−231 mV) demonstrating that the Ni_10_Mo nanoparticles as well as the right ratio of Ni_10_Mo and MoO_2_ are crucial for the HER activity. Furthermore, the overpotential remained stable over the tested time with Ni_10_Mo/MoO_2_@Ni-foam-2h, while it increased at MoO_2_@Ni-foam with a degradation rate of −8 mV/h. To deliver a current density of −10 mA/cm^2^ Ni_10_Mo/MoO_2_@Ni-foam-2h, MoO_2_@Ni-foam and Ni-foam exhibited an overpotential of −14, −22, and −185 mV, respectively.

**Figure 6:**
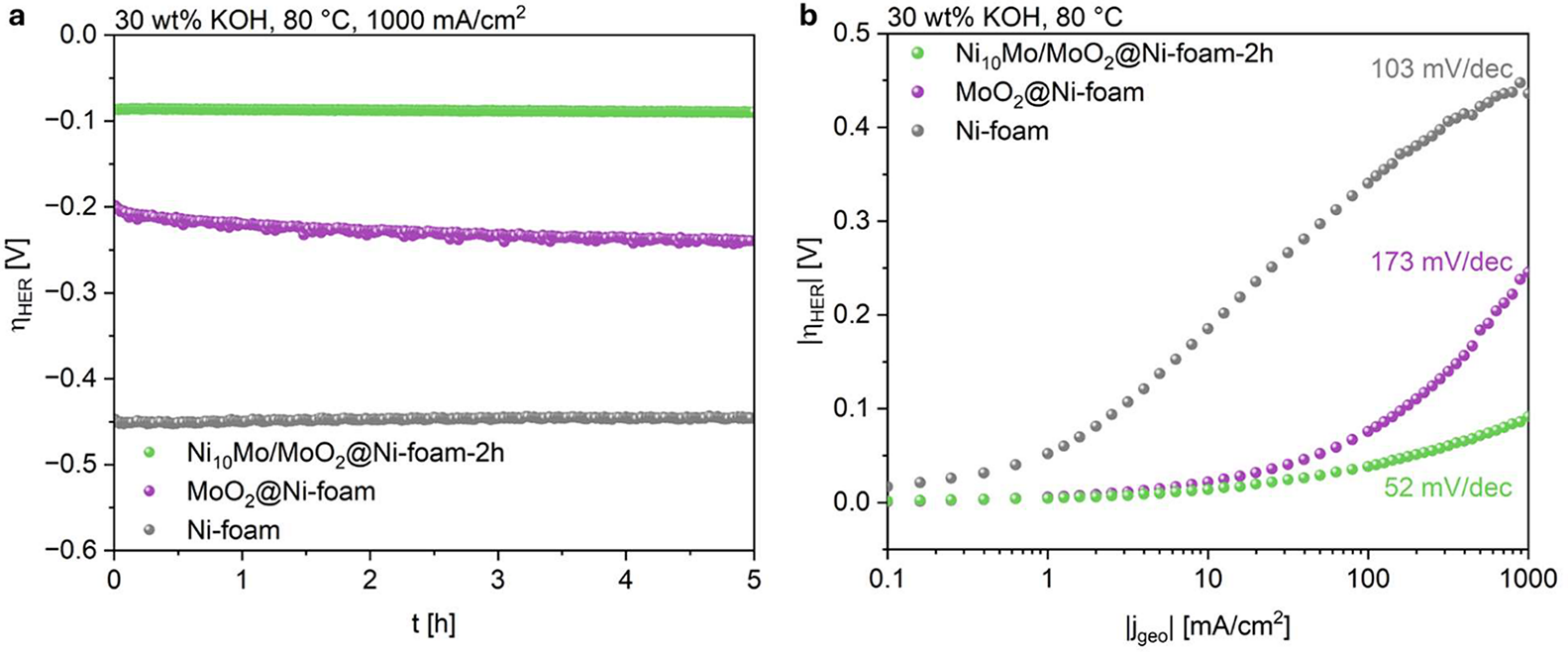
(a) Galvanostatic measurement and (b) Tafel plot of Ni_10_Mo/MoO_2_@Ni-foam-2h, MoO_2_@Ni-foam, and Ni-foam in 3EA.

The double-layer capacitance (*C*_DL_) was determined by cyclic voltammetry (CV). The measured *C*_DL_ values for Ni_10_Mo/MoO_2_@Ni-foam-2h, MoO_2_@Ni-foam and Ni-foam were 0.45, 0.18, and 0.0014 F, respectively. For comparison, the *C*_DL_ of smooth nickel is reported to be approximately 20 ± 5 μF/cm^2^.^44^ Based on this specific capacitance, the electrochemically active surface areas (ECSA) of Ni_10_Mo/MoO_2_@Ni-foam-2h, MoO_2_@Ni-foam and Ni-foam were calculated to be 22,000, 9000 and 70 cm^2^, respectively. The high C_DL_ and corresponding ECSA of Ni_10_Mo/MoO_2_@Ni-foam-2h are consistent with its microcuboid morphology, which provides an extensive electrochemically accessible surface. To distinguish between geometric and intrinsic catalytic effects, the polarization curves were normalized to the respective ECSA (Figure S14). After this normalization, Ni_10_Mo/MoO_2_@Ni-foam-2h still exhibits the highest intrinsic activity across the investigated overpotential range (η_HER_ = 0 to −100 mV vs RHE). For example, at an overpotential of −90 mV, the ECSA-normalized current density of Ni_10_Mo/MoO_2_@Ni-foam-2h is 1.3 times higher than that of bare Ni-foam and about 3 times higher than that of MoO_2_@Ni-foam. The fact that MoO_2_@Ni-foam itself possesses a much larger surface area than Ni-foam (9000 vs 70 cm^2^) yet remains less active per ECSA underscores that surface enlargement alone cannot account for the observed performance. It should also be noted that the ECSA derived from the double-layer capacitance represents the electrolyte-accessible area, which does not necessarily equal the number of catalytically active sites. We assume that the HER predominantly occurs at the Ni_10_Mo/MoO_2_ interfaces, which constitute only a fraction of the total accessible surface. Hence, the ECSA-based normalization likely overestimates the real active area, implying that the intrinsic activity of Ni_10_Mo/MoO_2_@Ni-foam-2h may, in fact, be even higher than suggested by the normalized data. The calculation of the *C*_DL_, ECSA and ECSA normalized current densities are provided in the Supporting Information (Table S2 and Figures S13, S14).

The Tafel slope was evaluated at current densities between 100 and 1000 mA/cm^2^, as the increase of overpotential at high current densities is relevant for industrial application. Ni_10_Mo/MoO_2_@Ni-foam-2h showed by far the lowest Tafel slope (52 mV/dec) compared with MoO_2_@Ni-foam (173 mV/dec) and Ni-foam (103 mV/dec) (Figure 6b). The substantially lower Tafel slope observed for Ni_10_Mo/MoO_2_@Ni-foam-2h reflects a genuine modification of the reaction energetics and rate-determining steps induced by the Ni–Mo alloy, rather than a purely geometric contribution, as a uniform increase in surface area would proportionally scale the exchange current density and would not affect the Tafel slope.

Besides the fact that the more active electrocatalyst shows a lower Tafel slope, it can provide insight on the rate determining step (RDS) of the HER. Under standard conditions Tafel slopes of 120, 40, and 30 mV/dec correspond to the Volmer, Heyrovsky, or Tafel step as RDS, respectively.^45^ The Tafel slopes of Ni_10_Mo/MoO_2_@Ni-foam-0.5h, Ni_10_Mo/MoO_2_@Ni-foam-2h, and Ni_10_Mo/MoO_2_@Ni-foam-3h were evaluated (Table 2 and Figure S9b) to identify the respective RDS. To confirm the kinetic validity of the Tafel analysis, we evaluated the Tafel slope as a function of current density (Figure S15), confirming that it remains constant within the relevant range. During the reduction process, molybdate is converted to MoO_2_, accompanied by the exsolution of Mo-containing nickel nanoparticles. Consequently, the reduction duration is expected to significantly influence both the catalytic activity and the Tafel slope. The Tafel slope for Ni_10_Mo/MoO_2_@Ni-foam-0.5h is above 120 mV/dec for current densities from 10 to 500 mA/cm^2^. After 0.5 h reduction only few Ni_10_Mo nanoparticles have formed on the surface, therefore, the migration of adsorbed hydrogen to Ni_10_Mo and the subsequent recombination to molecular hydrogen at the metallic site is slow. Further water activation at MoO_2_ is hindered and becomes rate limiting. Beyond that, incomplete Mo^6+^-reduction to Mo^4+^ prevents efficient water activation at the oxide. The Tafel slope for Ni_10_Mo/MoO_2_@Ni-foam-3h is approximately 30 mV/dec at low current densities, corresponding to the Tafel step being rate determining. With increasing current density, the slope rises gradually. This results in a slope of 54 mV dec^–1^ in the intermediate range (10 to 100 mA/cm^2^) and even higher values above 100 mA/cm^2^. After 3 h reduction the nanoparticles became larger and more densely packed, resulting in increased coverage of the MoO_2_ surface. This leads to the Tafel step being the RDS at low current densities but the Volmer step being the RDS at high current densities because of the low availability of MoO_2_. Meanwhile, the ratio of Ni_10_Mo and MoO_2_ is optimized with a 2 h reduction, resulting in a low Tafel slope in the current density range of 10 to 1000 mA/cm^2^ suggesting that the Tafel step is rate-limiting.

**Table 2:** Tafel Slopes of Ni_10_Mo/MoO_2_ with Different Reduction Durations for Intermediate and High Current Densities

	Tafel slope [mV/dec]		
current density range [mA/cm^2^]	Ni_10_Mo/MoO_2_@Ni-foam-0.5h	Ni_10_Mo/MoO_2_@Ni-foam-2h	Ni_10_Mo/MoO_2_@Ni-foam-3h
10–100	126	25	54
100–500	129	46	137

These findings demonstrate that the reduction time is strongly related with kinetics by altering the catalyst′s surface composition and morphology. This observation is consistent with the findings of Luo et al.,^24^ who reported that at Ni_*x*_Mo_*y*_/MoO_*x*_ heterostructures, water dissociation (Volmer step) predominantly occurs at the MoO_*x*_ component, whereas the Ni_*x*_Mo_*y*_ sites facilitate hydrogen adsorption and desorption (Tafel step). The structural refinement revealed that the metallic nanoparticles correspond to a Ni_10_Mo alloy rather than the often-reported Ni_4_Mo. Both alloys, however, represent Ni-rich Ni–Mo phases in which Mo atoms occupy minority lattice sites and similarly modulate the electronic structure of the active Ni centers.^46^ Therefore, the local catalytic motifs and the associated HER mechanism are expected to be essentially the same for Ni_4_Mo and Ni_10_Mo. The key role of Mo is to tune the electronic properties of Ni and thereby optimize hydrogen adsorption and desorption. This finding indicates that the formation of a specific Ni_4_Mo phase is not a prerequisite for high catalytic activity. Rather, the decisive factor is the presence of Ni-rich Ni–Mo nanoparticles electronically coupled to the conductive MoO_2_ matrix, which ensures efficient charge transfer and enhanced intrinsic HER kinetics. In addition, the spatial separation of these active sites suggests a spillover mechanism.^47^ Here, adsorbed hydrogen atoms generated during water dissociation on the MoO_2_ surface migrate to adjacent Ni_10_Mo domains, where they recombine to form molecular hydrogen. This transfer process between the two phases facilitates a more efficient reaction. The MoO_2_/Ni_10_Mo interface emerges as a key site for HER activity. Although it might be expected that longer reduction times–resulting in larger exsolved nickel–molybdenum particles and, thus, more interfacial area–would enhance the HER activity, the observed optimum at 2 h suggests a more complex correlation. The HER efficiency is influenced not only by the size of the interface but also by the balanced presence of MoO_2_, which facilitates water dissociation, and Ni_10_Mo, which promotes hydrogen recombination. Hence, the enhanced performance arises from both the individual catalytic roles of MoO_2_ and Ni_10_Mo as well as their cooperative interaction via hydrogen spillover. We hypothesize a schematic representation of this mechanism as provided in Figure 7.

**Figure 7:**
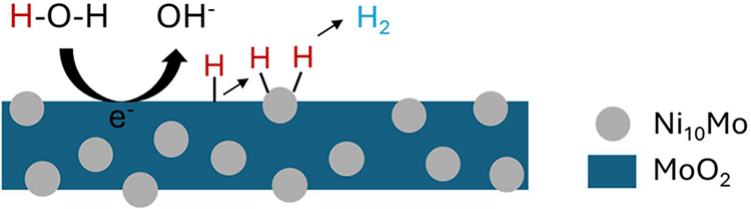
Schematic illustration of the HER on Ni_10_Mo/MoO_2_.

To assess whether electronic transport across the Ni foam-Ni_10_Mo/MoO_2_ interface influences the HER kinetics, galvanostatic electrochemical impedance spectroscopy (GEIS) was performed at various current densities (−10, −100, −300, and −500 mA/cm^2^) in 30 wt % KOH at 80 °C for all Ni_10_Mo/MoO_2_@Ni-foam-*x*h samples (*x* = 0.5, 2, 3). The corresponding Nyquist plots are shown in Figure S16. For Ni_10_Mo/MoO_2_@Ni-foam-0.5h and Ni_10_Mo/MoO_2_@Ni-foam-3h, the spectra exhibit a single prominent semicircle, attributable to the charge-transfer resistance (*R*_ct_) of the HER. No distinct high-frequency feature that would indicate an additional interfacial process between the Ni-foam substrate and the Ni_10_Mo/MoO_2_ coating is observed. The Ni_10_Mo/MoO_2_@Ni-foam-2h electrode, which displays the highest activity, exhibits the smallest *R*_ct_ across all current densities and only a weak shoulder at high frequencies at intermediate currents. This small feature suggests the onset of a second interfacial process that becomes detectable only because *R*_ct_ is significantly reduced for this highly active electrode. The high-frequency intercept (*R*_s_) remains nearly identical for all electrodes and current densities, confirming that no additional ohmic losses arise from the catalyst-substrate interface. These results demonstrate that the electronic coupling between the metallic Ni-foam substrate and the Ni_10_Mo/MoO_2_ coating is highly efficient, and that the overall impedance is dominated by the HER charge-transfer process. Accordingly, the RDS inferred from the Tafel slopes can be attributed to intrinsic catalytic processes rather than limited by interfacial electron transport.

To address the stability of Ni_10_Mo/MoO_2_ during shutdown, we quantified Mo dissolution under open-circuit conditions following a protocol adapted from Wang et al.^48^ The catalyst showed negligible Mo loss at OCP, confirming its stability in this regime. Detailed methodology and data are reported in the Supporting Information (Table S3). The stability under dynamic operating conditions, as relevant for electrolysis systems driven by renewable energy, was investigated by cycling between low and high current densities. The Ni_10_Mo/MoO_2_ catalyst showed stable performance during the test. Details of the experimental procedure and the corresponding data are provided in the Supporting Information (Figure S17).

A direct comparison of HER activities reported in the literature is often complicated by substantial differences in experimental conditions. While most studies on Ni–Mo-based catalysts are conducted in 1 M KOH at room temperature, the present work focuses on highly alkaline conditions (30 wt % KOH) at elevated temperature (80 °C), which are significantly closer to industrial alkaline electrolysis. To enable a meaningful comparison, Table S7 summarizes reported HER performances of non-PGM catalysts measured under similarly harsh conditions in half-cell configurations. As shown in this comparison, only few studies address such conditions. Within this limited data set, the Ni_10_Mo/MoO_2_@Ni-foam-2h catalyst demonstrates a favorable HER performance, combining a low overpotential at high current density with a low Tafel slope, underscoring its potential for alkaline water electrolysis under industrially relevant conditions.

The Ni_10_Mo/MoO_2_ catalyst was also investigated in a single-cell test at 80 °C with 30 wt % KOH as electrolyte. The catalyst coated on Ni foam and Ni fiber felt were used as cathode and a Raney-Ni mesh as anode. For comparison, a commercial Pt@C electrode was tested as cathode in the same setup. Figure 8a shows the galvanostatic measurement at 1 A/cm^2^ in the full cell setup. The cell with the Ni_10_Mo/MoO_2_@Ni-felt cathode exhibits a very low voltage of 1.71 V at this current density. The voltage remains stable over the measured time of 12 h and is significantly lower than the voltage of the cell with Ni_10_Mo/MoO_2_@Ni-foam (1.93 V after 12 h) and even Pt@C (1.78 V after 12 h) as cathode. This demonstrates that under the given experimental conditions, the Ni_10_Mo/MoO_2_@Ni-felt cathode enables a lower cell voltage compared to the benchmark Pt@C cathode, highlighting its high HER activity. It also shows that the choice of the porous transport layer is crucial. The enhanced performance of the Ni-felt substrate compared to the Ni-foam is due to two factors: (I) decreased thickness of the Ni-felt (0.5 mm) compared to the Ni-foam (1.6 mm) which leads to a lower electrical resistance and (II) a higher surface area of the Ni-felt which can offer more active centers for HER. Figure 8b shows the polarization curves of the three different cell measurements. As expected, the curve of the Ni_10_Mo/MoO_2_@Ni-felt cell runs at the lowest voltages and the slope (238 mΩ·cm^2^) is ∼1.5 times smaller than the one of the Ni_10_Mo/MoO_2_@Ni-foam cell (366 mΩ·cm^2^) and ∼1.3 times smaller the one of the Pt@C cell (309 mΩ·cm^2^). In all three cases, the polarization curves before and after the galvanostatic measurement at 1 A/cm^2^ (named initial and final) are almost identical and the cell voltage at a current density of 1 A/cm^2^ is even slightly lower after it. The high frequency resistance (HFR) at the beginning of test (BOT) and end of test (EOT) of the three investigated cell configurations is summarized in Table S4. At a cell voltage of 1.0 V, all three cells exhibit stable HFR values. Among them, the Pt@C cell shows the lowest ohmic resistance, followed by the Ni_10_Mo/MoO_2_@Ni-felt cell. Although the Ni_10_Mo/MoO_2_@Ni-felt cell has a slightly higher ohmic resistance than the Pt@C cell, it still demonstrates an enhanced overall electrochemical performance. This suggests that the Ni_10_Mo/MoO_2_ catalyst possesses highly efficient reaction kinetics and high intrinsic catalytic activity, compensating for minor resistive losses. At a current density of 700 mA/cm^2^ all three cells show similar HFR BOT values. But while at the EOT the HFR has increased for the Ni_10_Mo/MoO_2_@Ni-foam cell, it decreased for the Ni_10_Mo/MoO_2_@Ni-felt and Pt@C cells. This suggests differing gas bubble and water management characteristics over time. It can be inferred that the mass transport conditions degrade in the Ni-foam structure, possibly due to less favorable wettability or pore clogging, whereas the Ni-felt and C-paper structures benefit from improved interface conditions such as wettability and efficient gas removal in zero-gap operation. Repetition measurements are provided in Figure S18.

**Figure 8:**
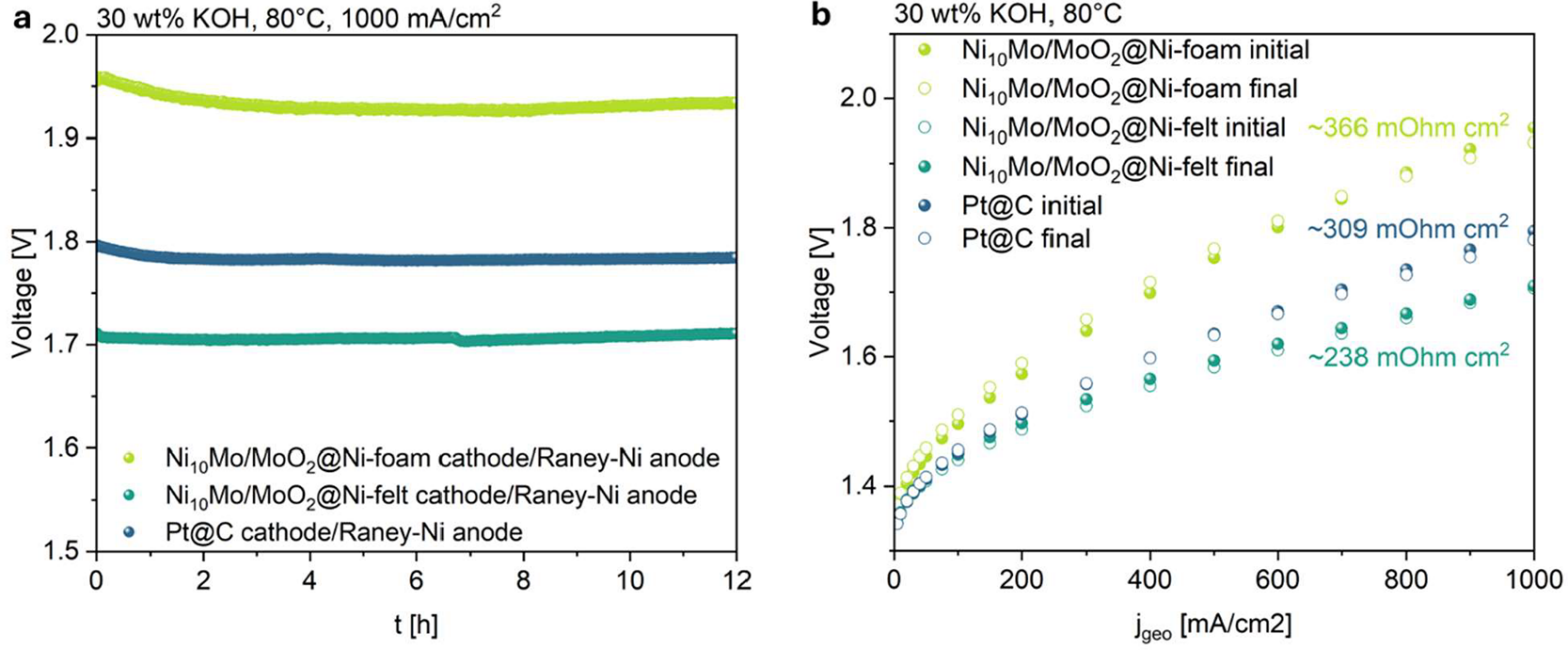
(a) Galvanostatic measurement and (b) polarization curves of Ni_10_Mo/MoO_2_@Ni-foam, Ni_10_Mo/MoO_2_@Ni-felt, and Pt@C in a single-cell setup.

Long-term durability is a crucial criterion for catalysts, particularly for industrial applications where extended operation is essential. To evaluate the catalyst’s stability, a 100-h durability test was conducted at 670 mA/cm^2^ in a single-cell setup (Figure 9a). Ni_10_Mo/MoO_2_@Ni-felt and NiFe layered double hydroxide on Ni felt (NiFe-LDH@Ni-felt) were used as the active layer on the cathode and anode side, respectively. The cell initially exhibited a low operating voltage of only 1.58 V. Over the course of 100 h, the voltage increased only marginally, reaching 1.61 V at the end of the test, indicating remarkable electrochemical stability under continuous operation. Polarization curves recorded before and after the durability test reveal only minor changes (Figure 9b). The polarization resistance remained low, with slope values of 187 and 202 mΩ·cm^2^, respectively. Similarly, the cell voltage at a current density of 1 A/cm^2^ increased only slightly from 1.64 to 1.67 V, further confirming the catalyst’s suitability for long-term performance. For this cell configuration, the Faradaic efficiency (FE) was determined for both HER at the cathode and the OER at the anode (Tables S5 and S6). FE values close to 100% indicate that the applied current is almost entirely utilized for the intended water-splitting reactions, with negligible side reactions occurring at either electrode.

**Figure 9:**
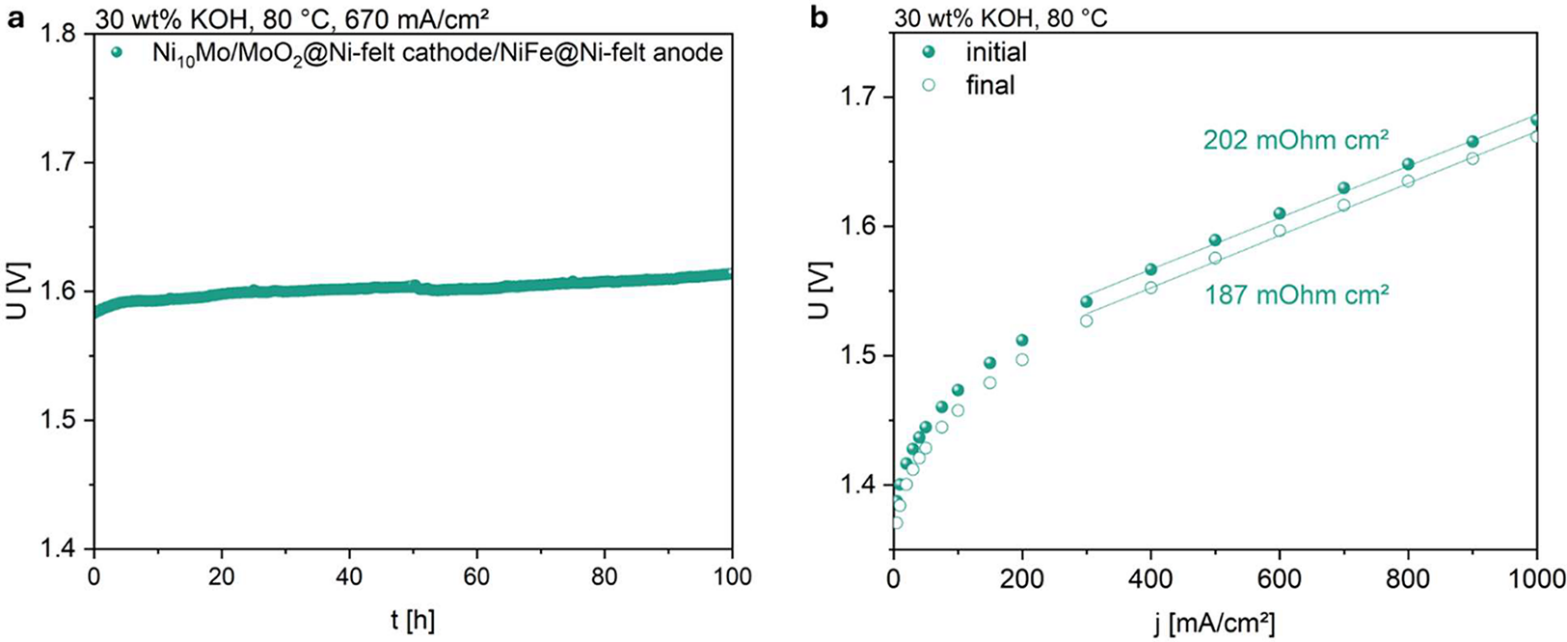
(a) 100-h durability test and (b) polarization curves of Ni_10_Mo/MoO_2_@Ni-felt before and after the test.

Post-testing SEM analysis (Figure S19) revealed partial morphological changes of the Ni_10_Mo/MoO_2_@Ni-felt cathode after the 100-h single-cell test. While the cuboid structure remains, their surface appears partially dissolved. These modifications can be attributed to local corrosion or surface restructuring processes occurring under electrochemical conditions. However, XRD patterns (Figure S20) recorded after the test still exhibit the same phases as before, indicating that the phase composition remains intact.

To evaluate the performance of the investigated cell, a comparison (Table S8) was conducted with reported cell configurations operating under similarly harsh conditions (highly concentrated alkaline electrolytes, elevated temperatures, high current densities). The tested cell exhibits an initial voltage of 1.58 V at a current density of 0.67 A/cm^2^, representing a lower cell voltage at a higher current density than most systems reported in the literature. Furthermore, the degradation rate of +0.3 mV/h over 100 h is up to an order of magnitude lower than those previously reported, which were often measured at much lower current densities. These findings highlight the strong potential of the Ni_10_Mo/MoO_2_ catalyst for industrial alkaline electrolysis, offering a favorable combination of high performance and long-term durability.

## 4 Conclusions

In summary, through the exsolution of Ni–Mo nanoparticles on MoO_2_ cuboids under reductive conditions an active HER catalyst was obtained. The material benefits from the combination of the MoO_2_ matrix with a large surface area and good electrical conductivity as active sites for fast water dissociation and the Ni_10_Mo alloy as active sites for hydrogen adsorption and recombination to molecular hydrogen. It was demonstrated that the reaction kinetics are heavily influenced by the reduction time by altering the catalyst′s surface composition and morphology. With a reduction time of 2 h the ratio of Ni_10_Mo and MoO_2_ is optimized regarding a sufficiently large interface for hydrogen spillover, resulting in short hydrogen migration pathways, as well as a balanced presence of the two phases as active sites for the different steps of the HER.

The Ni_10_Mo/MoO_2_@Ni-foam-2h catalyst demonstrates an overpotential of −89 mV at a current density of −1 A/cm^2^. Furthermore, the catalyst supported on Ni fiber felt outperformed a commercial Pt–C cathode in a full cell test. In comparison to previously reported alkaline electrolysis systems, it demonstrates a favorable combination of high current density, low cell voltage, and remarkable long-term stability. These results highlight the significant activity of the catalyst as well as its suitability for industrial applications, given the testing under industrially relevant conditions.

Detailed analysis of the catalyst suggests Ni_10_Mo nanoparticles at MoO_2_ as active species, rather than Ni_4_Mo/MoO_2_. Previous publications from other groups may have obtained similar materials but were not looking at the nanoscale deeply to see the details of the nanostructured electrocatalyst or were simply not aware of exsolution like processes as described here.

Beyond its demonstrated efficiency for HER in AWE, the Ni_10_Mo/MoO_2_ catalyst also has potential for other electrochemical applications. Other authors have reported similar materials as highly active catalysts for the oxygen evolution reaction (OER),^8–11,13,20,21,27,49,50^ as HER catalysts in anion exchange membrane water electrolyzers (AEMWE)^25,51,52^ and hydrogen oxidation reaction catalysts in anion exchange membrane fuel cells (AFC).^53–55^ The possibility to facilitate both HER and OER, to use it in AEMWE and AFC enhances its value and expands its applicability in next-generation hydrogen technologies. This makes Ni_10_Mo/MoO_2_ a promising versatile and scalable electrocatalyst for a sustainable hydrogen economy.

## Supplementary Material



## Notes

The Supporting Information is available free of charge at https://pubs.acs.org/doi/10.1021/acscatal.5c05933. The research data supporting this publication can be accessed at: https://doi.org/10.17630/5c14e21c-3abd-42ea-90d6-829f02317d3d

Additional XRD pattern, SEM images, HR-TEM analysis, XPS data, electrochemical data, comparison with literature and Faradaic efficiency measurements (PDF)
