# Supplementary material for: Nickel–Molybdenum Nanoparticles Anchored on Molybdenum Oxide as High-Performance Electrocatalyst for Hydrogen Production in Alkaline Water Electrolysis
Source: ACS Catal. 2026 Feb 15;16(5):4399–409. doi: 10.1021/acscatal.5c05933 (PMC12973263; doi:10.1021/acscatal.5c05933)
Supplement: Supplementary file 1 [file cs5c05933_si_001.pdf]

Nickel-Molybdenum Nanoparticles Anchored on Molybdenum Oxide as a High-Performance Electrocatalyst for Hydrogen Production in Alkaline Water Electrolysis

Anna K. Müller[1], Stefan Loos[1,*], Christian I. Bernäcker[1], Aaron Naden[2], Felix Heubner[1], Thomas Weißgärber[1,3]

[1] Fraunhofer Institute for Manufacturing Technology and Advanced Materials IFAM, Winterbergstraße 28, 01277 Dresden, Germany

[2] School of Chemistry, University of St Andrews, St Andrews KY16 9ST, U.K.

[3] Faculty Mechanical Engineering, Institute of Material Science, Chair Powder Metallurgy, TUD Dresden University of Technology, 01062 Dresden, Germany

Corresponding Author

* Stefan Loos – Fraunhofer Institute for Manufacturing Technology and Advanced Materials IFAM, Winterbergstraße 28, 01277 Dresden, Germany.

E-mail: stefan.loos@ifam-dd.fraunhofer.de

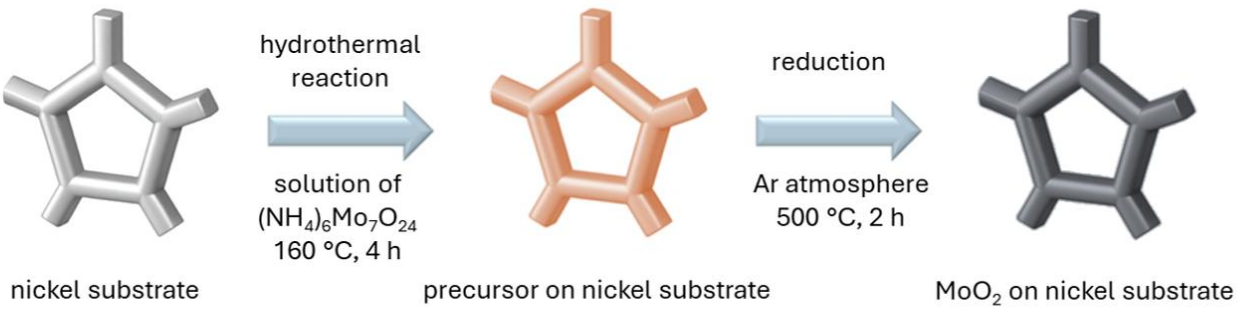

Figure S1. Schematic illustration of the MoO_2@Ni-foam synthesis.

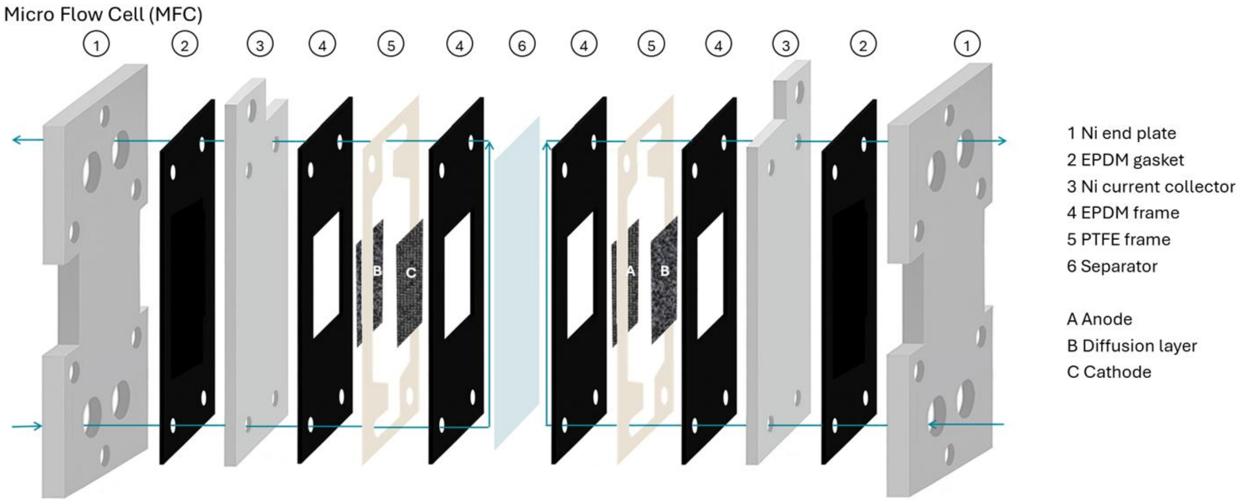

Figure S2. Schematic illustration of the Micro Flow Cell used for single-cell test.

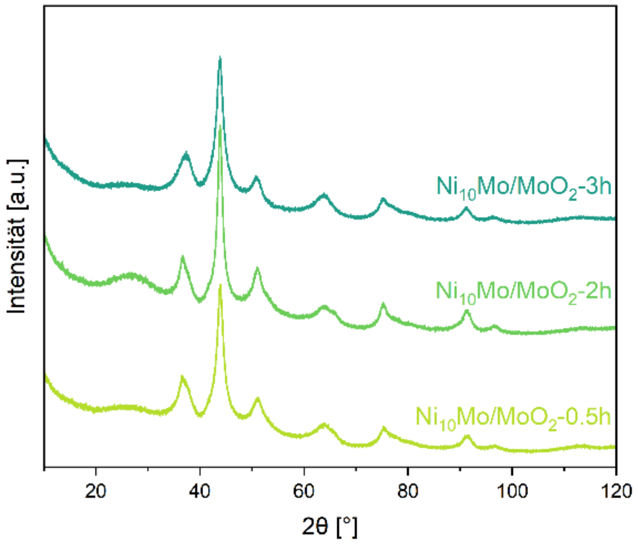

Figure S3. X-ray powder diffraction pattern of $Ni_{10}Mo/MoO_2$-0.5h, $Ni_{10}Mo/MoO_2$-2h and $Ni_{10}Mo/MoO_2$-3h.

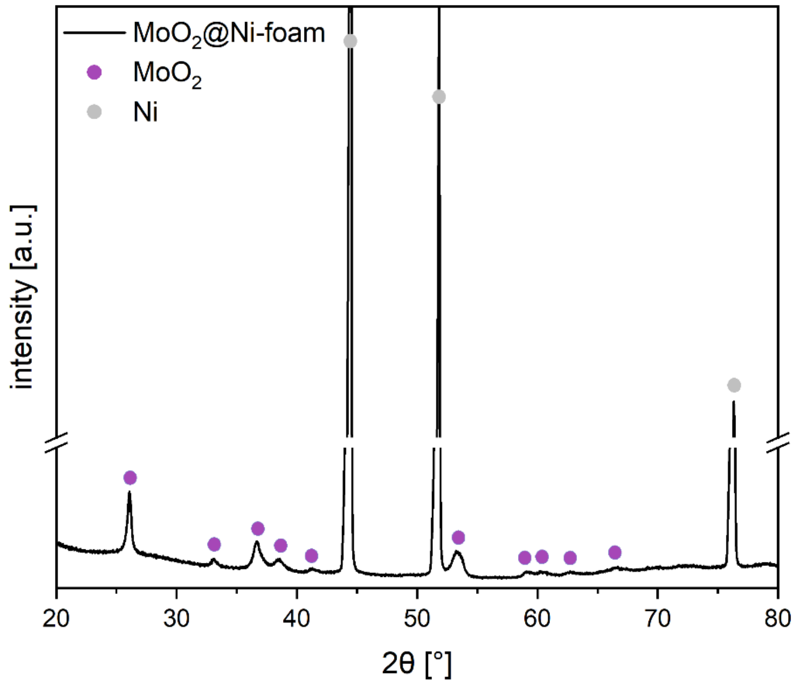

Figure S4. X-ray powder diffraction pattern of MoO_2@Ni-foam.

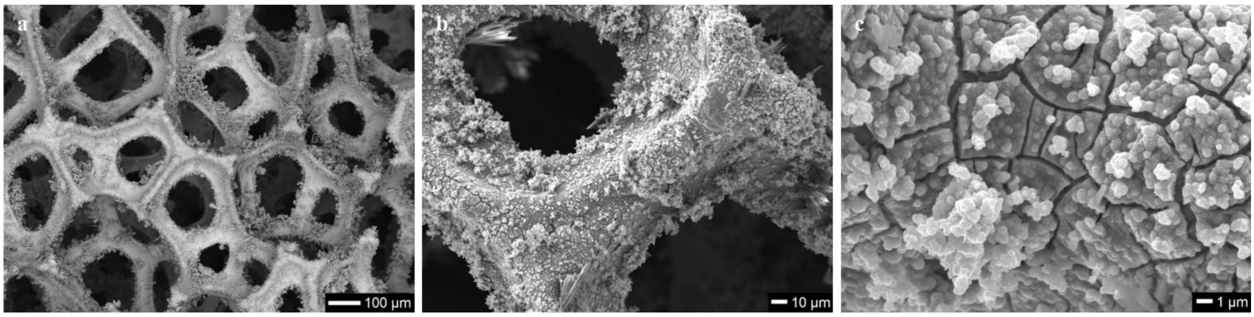

Figure 5. SEM images of MoO_2@Ni-foam.

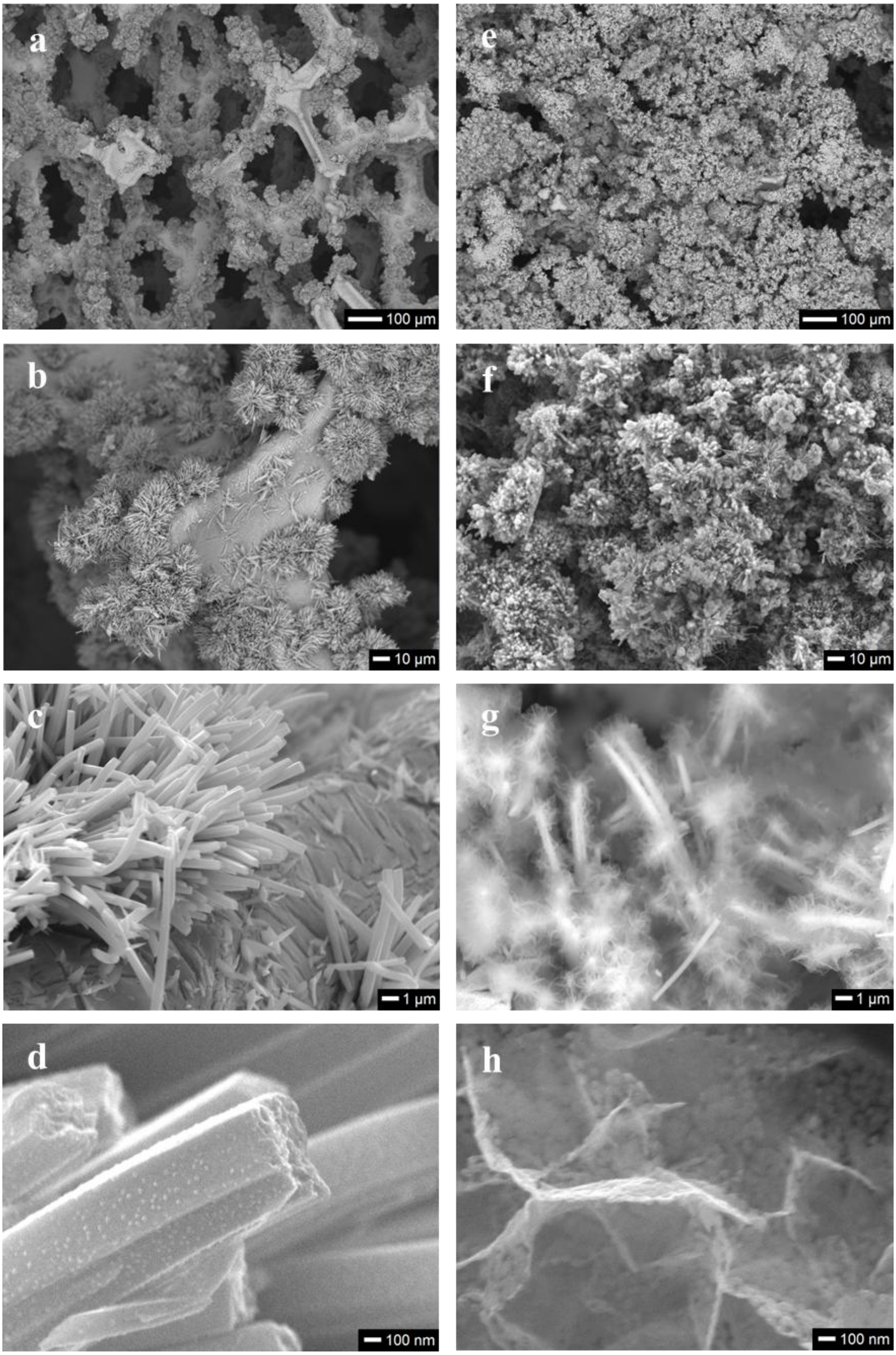

Figure S6. SEM images of $Ni_{10}Mo/MoO_2$@Ni-foam-0.5h (a-d) and $Ni_{10}Mo/MoO_2$@Ni-foam-3h (e-f).

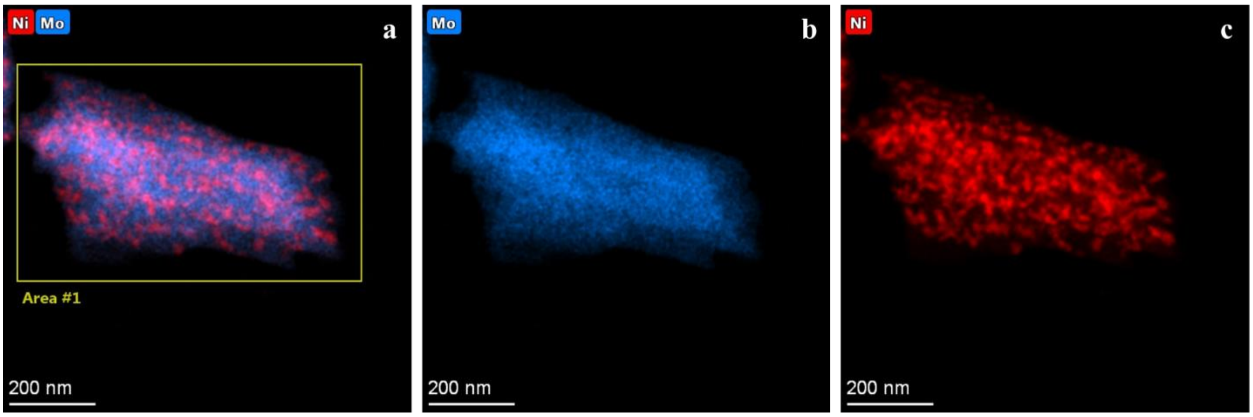

Figure S7. TEM-EDS mapping of a micro cuboid with nanoparticles (a), individual mapping of Mo (b) and Ni (c)

The *d*-spacings of several (hkl) planes were determined by HR-TEM at multiple spots of the sample and the corresponding lattice parameters were calculated using the following equation:

$$a = d \cdot \sqrt{h^2 + k^2 + l^2} \qquad \text{(eq. S1)}$$

The Mo content was then derived via Vegard's law:

$$x_{Mo} = \frac{a - 3.5266}{0.00426} \qquad \text{(eq. S2)}$$

The results are summarized in Table S1. The average Mo content obtained from HR-TEM was 7.6 ± 8.2 at%. However, the individual measurements show large deviations. In several cases, the calculated lattice parameter a was even smaller than that of pure Ni (3.5266 Å), corresponding to non-physical negative Mo contents. In these cases, an Mo content of 0 at% was assumed. The large deviation reflects the significantly higher uncertainty in lattice parameter determination via HR-TEM.

Table S1. *d*-spacing measured for (hkl) planes and calculated lattice parameter *a* and Mo content x_{Mo}

**hkl plane**	***d*** **[Å]**	$a_{calculated}$ **[Å]**	x_{Mo} **[at%]**	$x_{Mo,average}$ **[at%]**
111	2.07	3.5853	13.8	7.6 ± 8.2
	2.08	3.6027	17.9	
	2.08	3.6027	17.9	
200	1.76	3.5200	0	
	1.77	3.5400	3.2	
	1.74	3.4800	0	
220	1.26	3.5638	8.7	
	1.26	3.5638	8.7	
	1.24	3.5073	0	
311	1.06	3.5156	0	
	1.05	3.4825	0	
	1.09	3.6151	20.8	

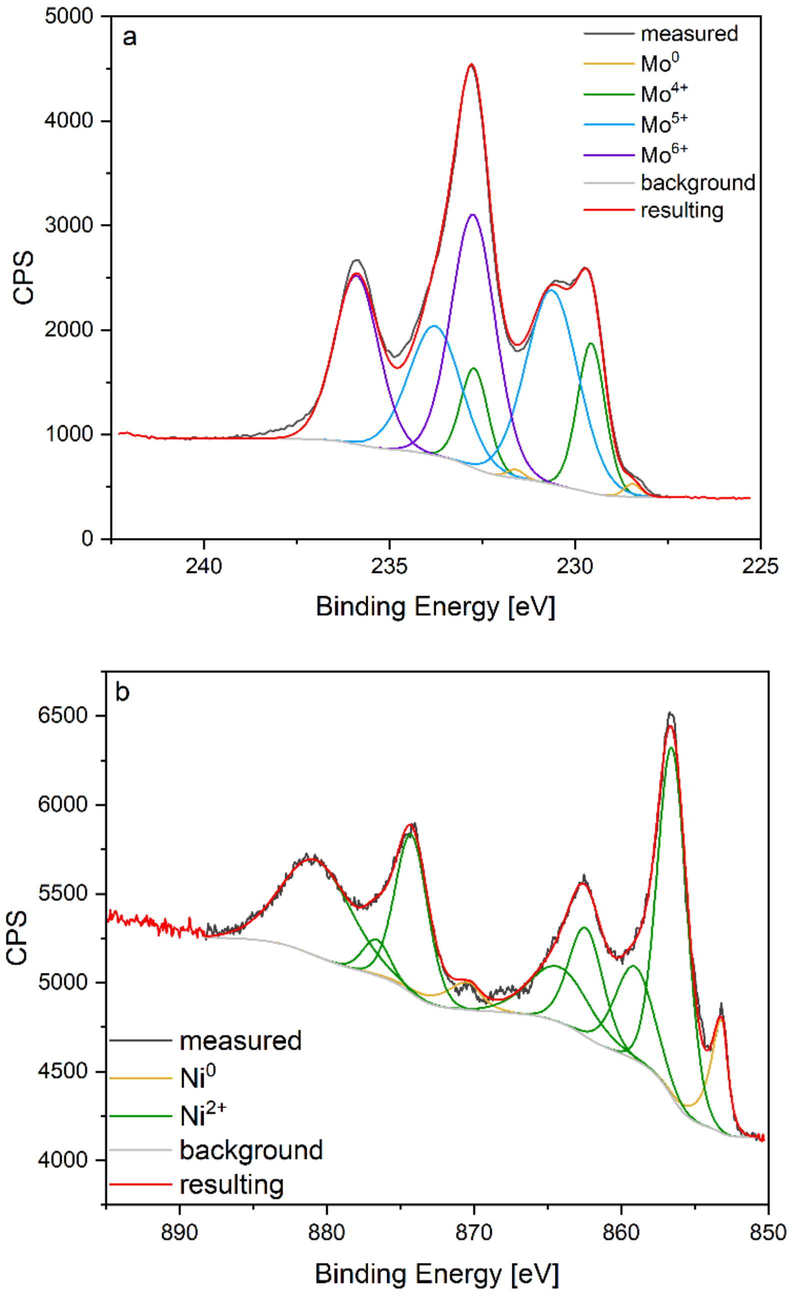

Figure S8. (a) Mo 3d and (b) Ni 2p XPS spectra of $Ni_{10}Mo/MoO_2$@Ni-foam-2h.

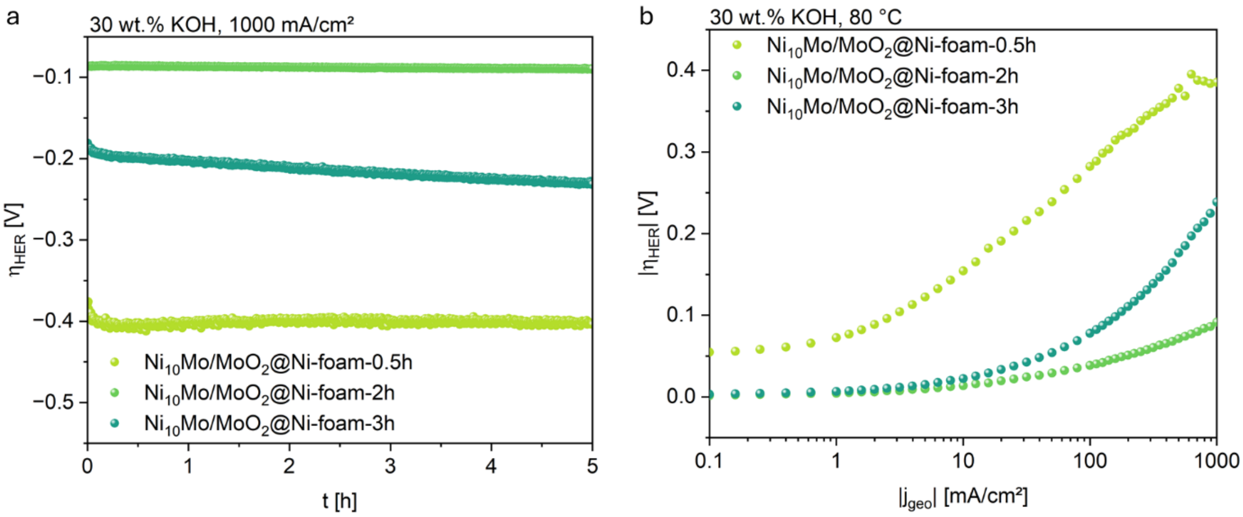

Figure S9. (a) Galvanostatic measurement and (b) Tafel plot of $Ni_{10}Mo/MoO_2$@Ni-foam-0.5h, $Ni_{10}Mo/MoO_2$@Ni-foam-2h and $Ni_{10}Mo/MoO_2$@Ni-foam-3h in 3EA.

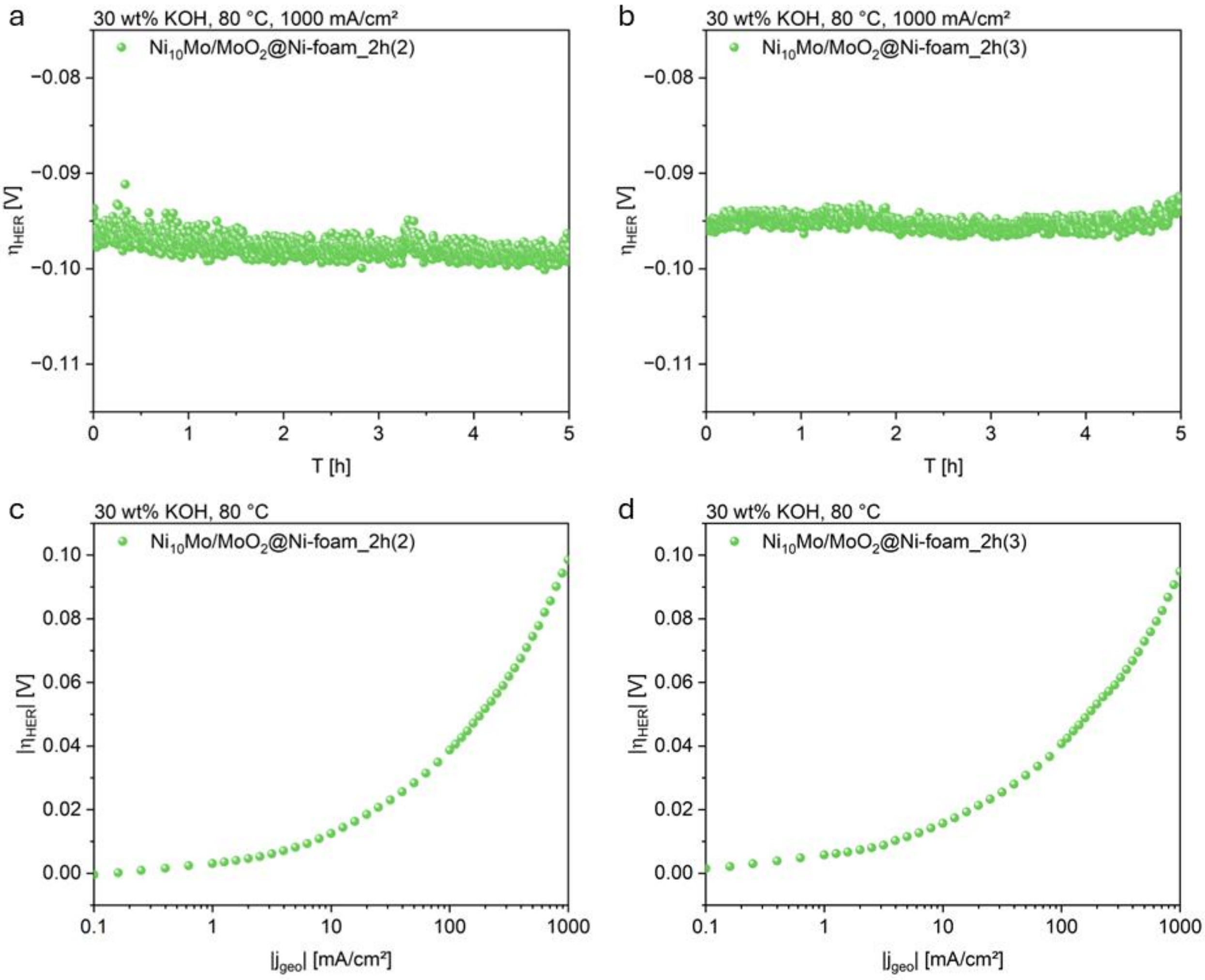

Figure S10. Repetitions of (a,b) galvanostatic measurement and (c,d) Tafel analysis of $Ni_{10}Mo/MoO_2$@Ni-foam-2h in 3EA.

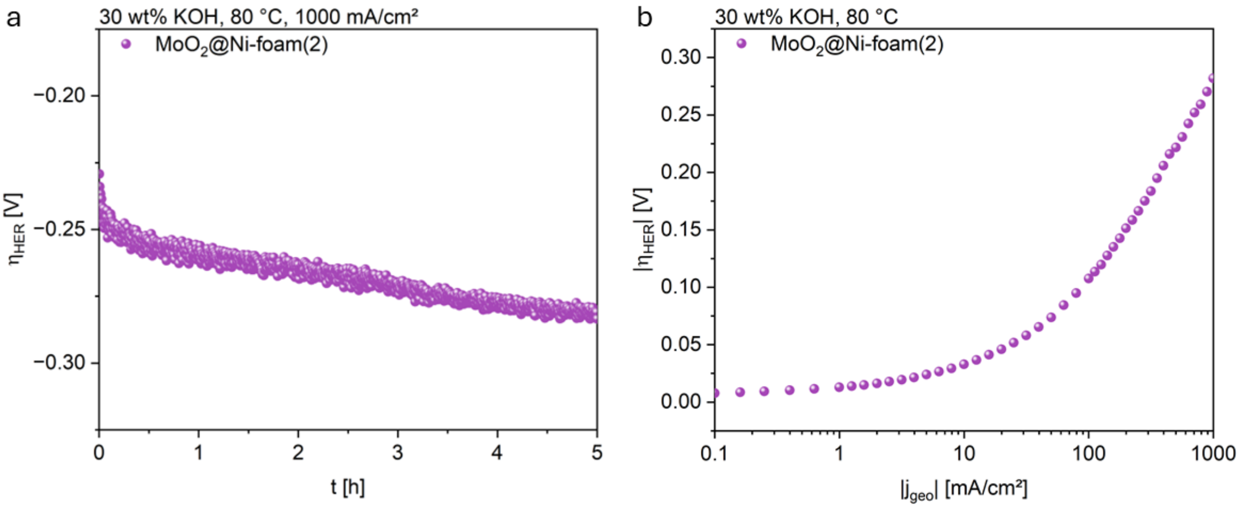

Figure S11. Repetition of (a) galvanostatic measurement and (b) Tafel analysis of MoO_2@Ni-foam in 3EA.

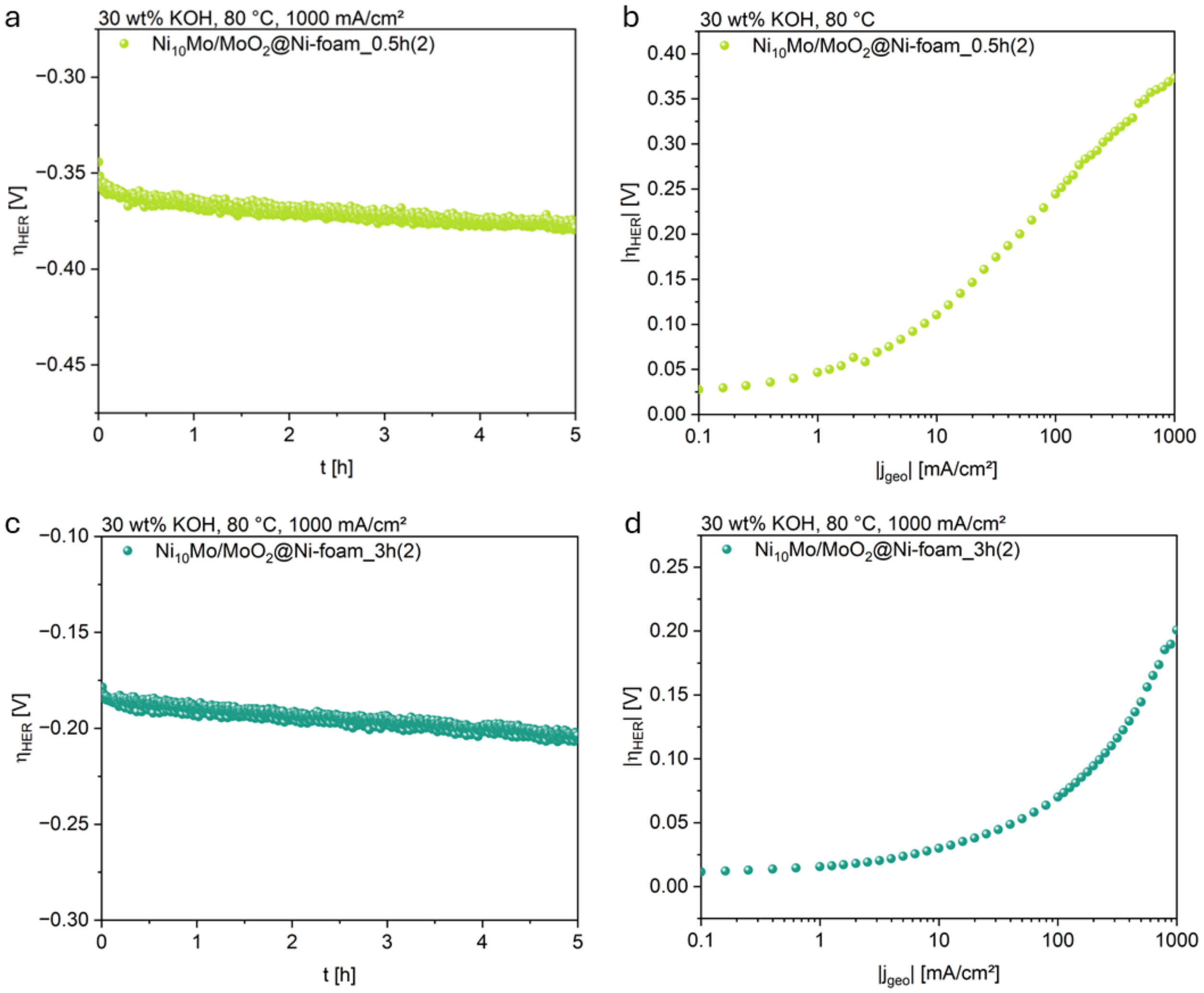

Figure S12. Repetitions of galvanostatic measurement and Tafel analysis of $Ni_{10}Mo/MoO_2$@Ni-foam-0.5h (a and b) and $Ni_{10}Mo/MoO_2$@Ni-foam-3h (c and d) in 3EA.

The electrochemically active surface area (ECSA) was estimated from the double-layer capacitance (C_{DL}), determined by cyclic voltammetry. Cyclic voltammograms were recorded over five cycles in the potential window of 0.175–0.275 V vs. RHE at various scan rates (Fig. S13 a-c), analogous to the procedure described by Zhang et al.[1]. The average current $\bar{I}$ at 0.225 V vs. RHE was calculated (eq. S1) and plotted as a function of the scan rate. The C_{DL} was obtained from the slope of the resulting linear fit (Fig. S13 d).

$$\bar{I} = \frac{I_a + |I_c|}{2} \qquad \text{(eq. S3)}$$

Where I_a and I_c are the cathodic and anodic current at 0.225 V vs. RHE, respectively.

The ECSA was then calculated using a specific capacitance value (C_{spec}) of 20 µF/cm², which corresponds to a smooth nickel surface in 30 wt% KOH at 60 °C (eq. S2).[2]

$$ECSA = \frac{C_{DL}}{C_{spec}} \qquad \text{(eq. S4)}$$

The ECSA-normalized current density was calculated the following relation (eq. S3):

$$j_{ECSA} = \frac{I}{ECSA} \qquad \text{(eq. S5)}$$

All calculated C_{DL} and ECSA values are listed in Table S1. The ECSA-normalized polarization curves are depicted in Fig. S14.

Table S2. Double-layer capacities (C_{DL}) and electrochemically active surface areas (ECSA) of $Ni_{10}Mo/MoO_2$@Ni-foam-2h, MoO_2@Ni-foam and Ni-foam

**Catalyst**	**C_{DL} [F]**	**ECSA [cm²]**
$Ni_{10}Mo/MoO_2$@Ni-foam-2h	0.45	22 000
MoO_2@Ni-foam	0.18	9 000
Ni-foam	0.0014	70

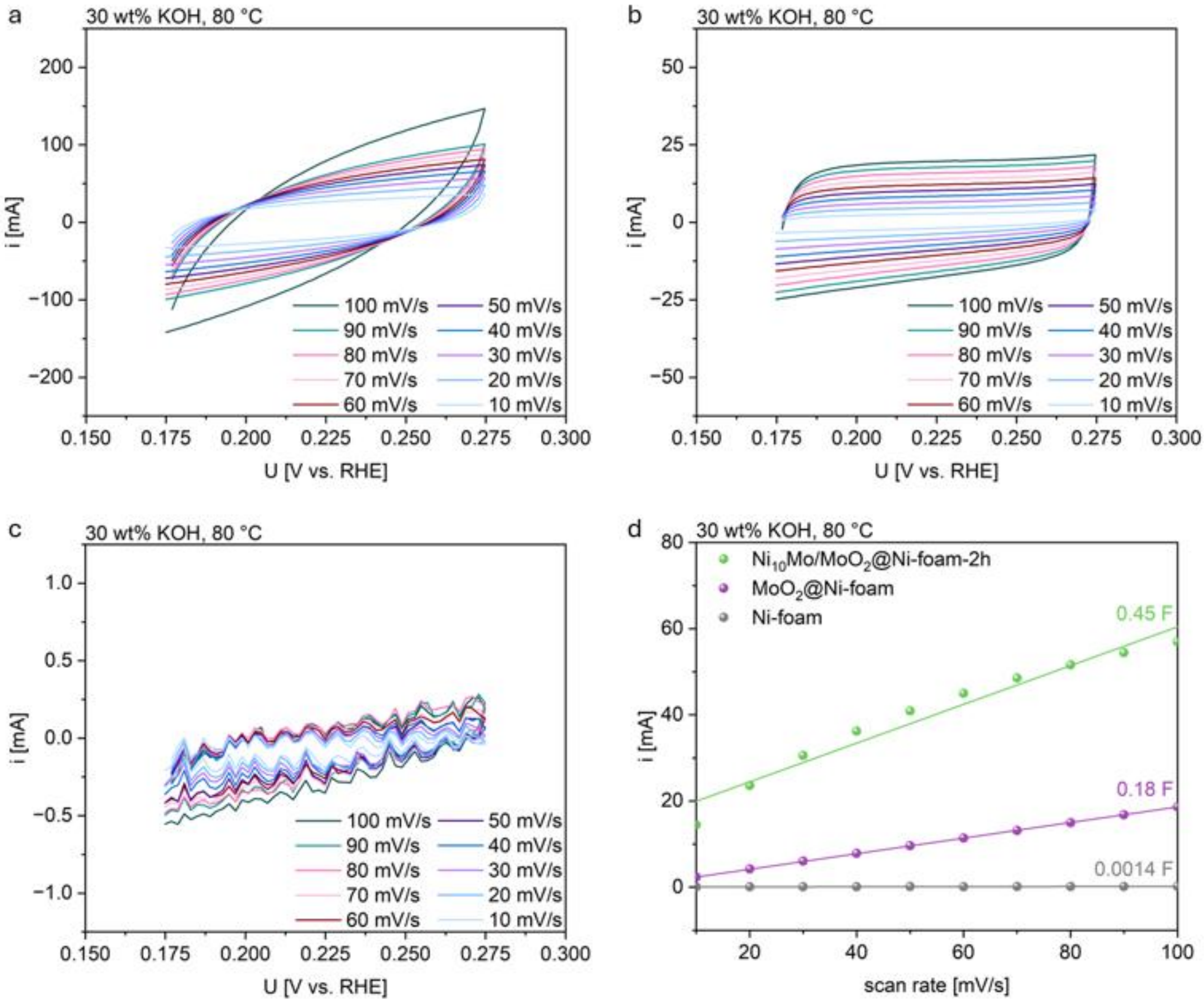

Figure S13. Cyclic voltammograms of $Ni_{10}Mo/MoO_2$@Ni-foam (a), MoO_2@Ni-foam (b) and Ni-foam (c). (d) The averaged current at 0.225 V vs. RHE plotted against the scan rate to determine the CDL from the linear fit.

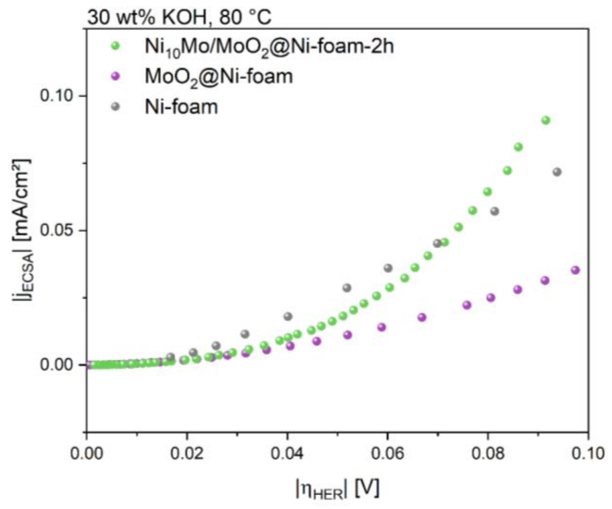

Figure S14. ECSA-normalized polarization curves for $Ni_{10}Mo/MoO_2$@Ni-foam, MoO_2@Ni-foam and Ni-foam.

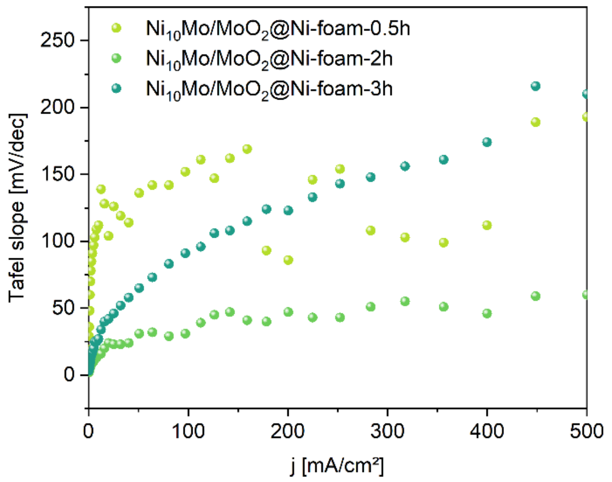

Figure S15. Tafel slope calculated over the range of three measurement points and plotted vs the average current density.

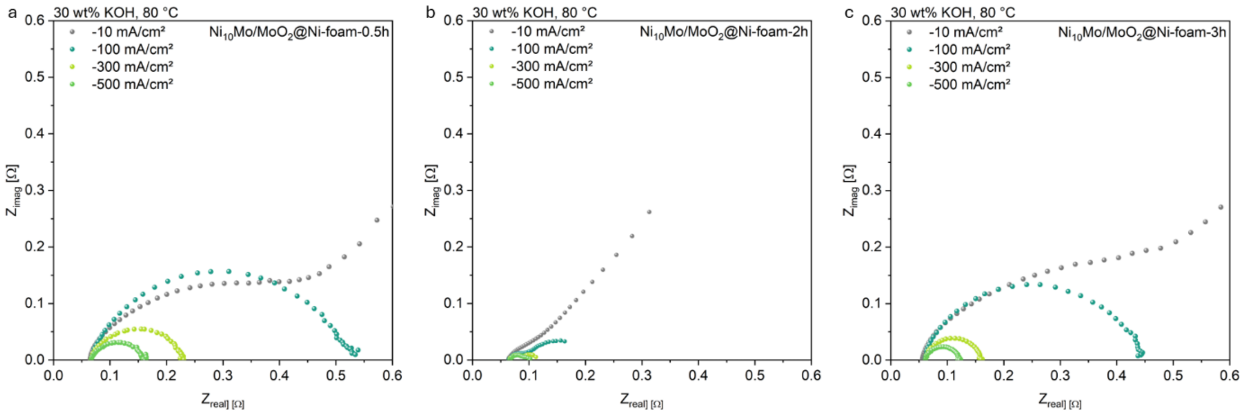

Figure S16. Galvanostatic electrochemical impedance spectra at -10, -100, -300, and -500 mA/cm² of $Ni_{10}Mo/MoO_2$@Ni-foam-xh samples (x = 0.5, 2, 3).

To evaluate the chemical stability of the $Ni_{10}Mo/MoO_2$ catalyst under open-circuit conditions, the $Ni_{10}Mo/MoO_2$@Ni-foam-2h electrode was exposed for 1 min each to OCP (0.12 V vs. RHE), 0.0 V, and -0.05 V vs. RHE, with three repetitions per potential. After each step, 20 ml of electrolyte (initially 0.6 l of 30 wt% KOH) were collected and analyzed by ICP-OES for dissolved Mo. To ensure quantitative comparability, the Mo concentrations were corrected for the sequential sample removals using a full mass-balance approach. The resulting net dissolution rates were converted to flux densities expressed as µmol s^{-1} cm^{-2} and are summarized in Table S3.

Table S3. Mo dissolution rates of $Ni_{10}Mo/MoO_2$@Ni-foam-2h at different potential

Potential	Mo dissolution rate [µmol s^{-1} cm^{-2}]
OCP	$2.6 \times 10^{-4} \pm 2.9 \times 10^{-3}$
0.00 V vs. RHE	$4.3 \times 10^{-3} \pm 4.6 \times 10^{-3}$
-0.05 V vs. RHE	$5.1 \times 10^{-4} \pm 6.8 \times 10^{-5}$

Within the precision of the ICP-OES measurements, Mo dissolution at OCP and small cathodic biases was negligible in the time scale investigated. These results confirm that the $Ni_{10}Mo/MoO_2$ composite remains chemically stable under shutdown-relevant conditions. The variations observed among repeated potential holds can be attributed to the analytical uncertainty of the ICP-OES method, minor differences in sampling, local mixing, or surface equilibration during the early stages. No systematic trend in Mo concentration was observed, confirming that the apparent changes represent random experimental scatter rather than progressive dissolution.

For accelerated stress testing (AST) the $Ni_{10}Mo/MoO_2$@Ni-foam-2h electrode was subjected to 100 current cycles, each consisting of alternating current densities of 10 mA/cm² and 1000 mA/cm² with a holding time of 5 min. This protocol was applied to evaluate the electrode stability under dynamic load conditions, relevant for electrolysis systems driven by renewable energy. Figure S17a shows the average HER overpotentials at 10 mA/cm² and 1000 mA/cm² for each cycle. The overpotential remains stable and below 100 mV at a current density of 1000 mA/cm² throughout the test. In addition, the Tafel slope (27 mV/dec between 10 mA/cm² and 100 mA/cm²) remains unchanged before and after AST. These results demonstrate that the $Ni_{10}Mo/MoO_2$ catalyst remains stable under the applied dynamic operating conditions.

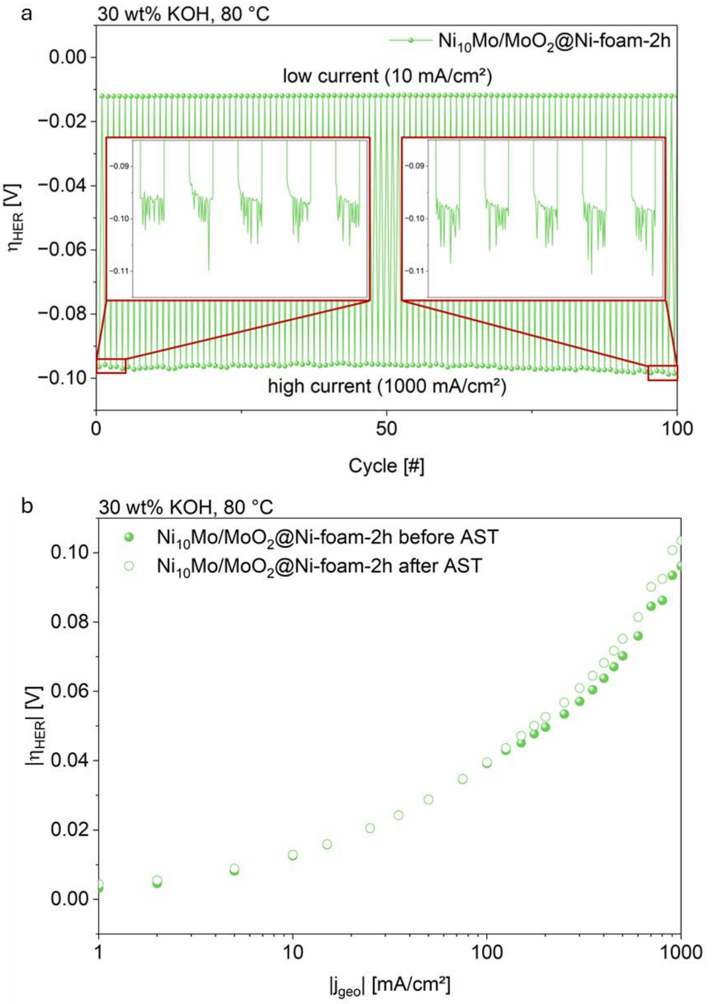

Figure S17. Cycling between high and low current density for AST (a) and Tafel plot before and after AST (b).

Table S4. High frequency resistance (HFR) at the begin of test (BOT) and end of test (EOT) of $Ni_{10}Mo/MoO_2$@Ni-foam, $Ni_{10}Mo/MoO_2$@Ni-felt and Pt@C in single-cell setup

**Cathode**	**HFR BOT [mΩ·cm²]**		**HFR EOT [mΩ·cm²]**	
	@1V	@700mA/cm²	@1V	@700mA/cm²
$Ni_{10}Mo/MoO_2$@Ni-foam	142	131	139	145
Pt@C	115	129	108	121
$Ni_{10}Mo/MoO_2$@Ni-felt	129	132	132	121

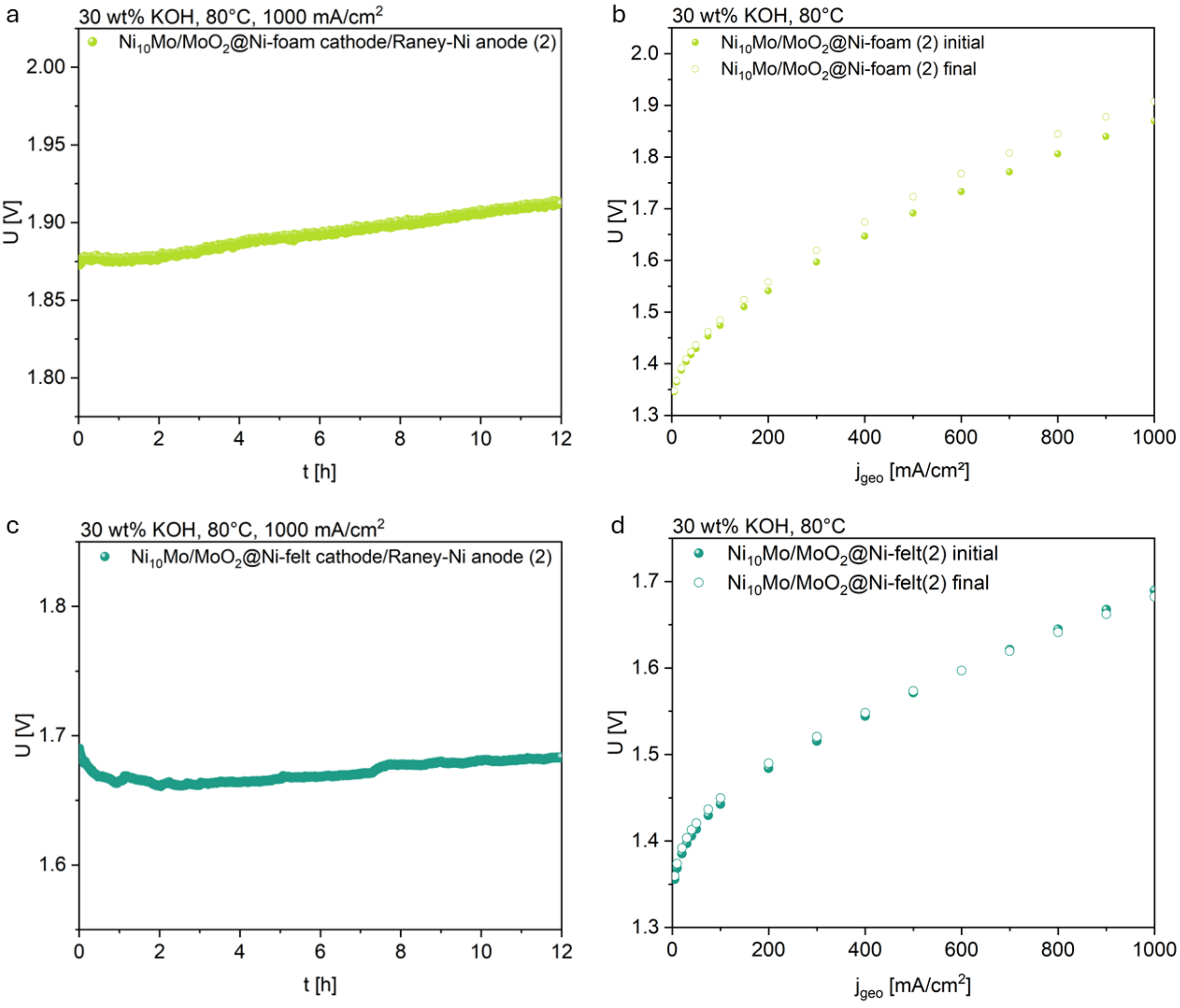

Figure S18. Repetitions of (a) galvanostatic measurement and (b) polarization curves of $Ni_{10}Mo/MoO_2$@Ni-foam (a and b) and $Ni_{10}Mo/MoO_2$@Ni-felt (c and d) in single-cell setup.

The Faradaic efficiency was determined in a single-cell setup by collecting and quantifying the hydrogen produced at the cathode and the oxygen produced at the anode. For this experiment $Ni_{10}Mo/MoO_2$@Ni-felt and NiFe-LDH@Ni-felt were used as the active layer on the cathode and anode side respectively. It was calculated by Faraday´s law (eq. S4) what time was required to produce 20 ml of hydrogen or 30 ml of oxygen at current densities of 50 mA/cm², 100 mA/cm² and 200 mA/cm².

$$t = \frac{F \cdot n \cdot z}{I} = \frac{F \cdot V_{theo} \cdot z}{I \cdot V_m} \qquad \text{(eq. S6)}$$

Where t is the time in s, F the Faraday constant (96485 As), n is the amount of produced gas in mol, z is the number of exchanged electrons (z=2 for hydrogen and z=4 for oxygen), I is the applied current in A, V_{theo} is the produced volume of hydrogen or oxygen and V_m is the molar volume (24.46 l/mol at 25 °C).

Each current density was applied for the corresponding calculated duration. The experiment was conducted three times for each current density and separately for the hydrogen and oxygen measurement. The Faradaic efficiency was calculated by dividing the experimentally measured hydrogen volume by the theoretically expected volume (eq. S5).

$$FE\ [\%] = \frac{V_{measured}}{V_{theo}} \cdot 100 \quad \text{(eq. S7)}$$

The Faradaic efficiency given in Table S1 and Table S2 is the average from three measurements with the corresponding standard deviation. The Faradaic efficiency close to 100 % proves that the HER and OER are the main processes at the cathode and anode respectively and barely any side reactions take place.

Table S5. Faradaic efficiency determined over produced hydrogen

j [mA/cm²]	t [s]	$V_{theo,H2}$ [ml]	$V_{measured,H2}$ [ml]	FE [%]
50	316	20.03	19; 20; 19	96.5 ± 2.9
100	158	20.03	20; 19.5; 20	99.0 ± 1.4
200	79	20.03	20; 20; 20	99.9 ± 0

Table S6. Faradaic efficiency determined over produced oxygen

j [mA/cm²]	t [s]	$V_{theo,O2}$ [ml]	$V_{measured,O2}$ [ml]	FE [%]
50	947	30.01	29; 29; 29	96.7 ± 0
100	473	29.98	29.5; 29.5; 29	97.8 ± 1.0
200	237	30.04	29.5; 29.5; 29.5	98.3 ± 0

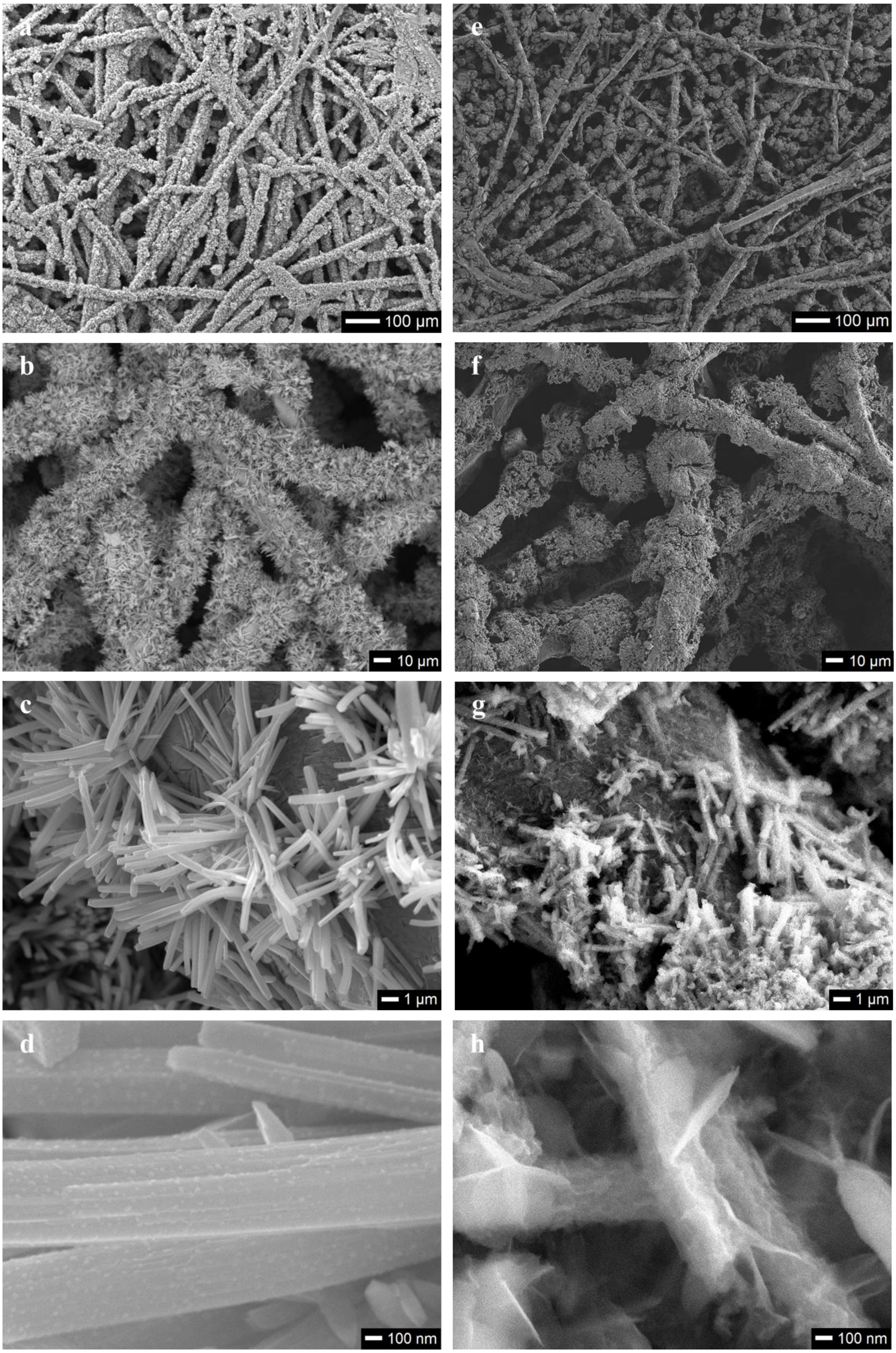

Figure S19. SEM images of $Ni_{10}Mo/MoO_2$@Ni-felt (a-d) before and (e-h) after the 100-hour single-cell test.

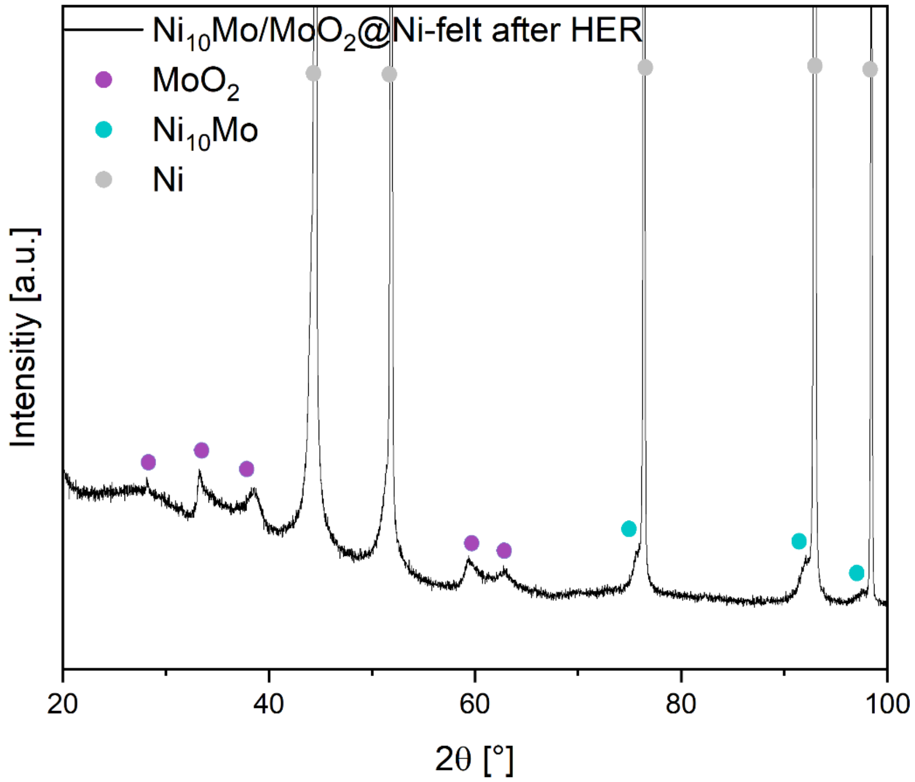

Figure S20. X-ray powder diffraction pattern of Ni10Mo/MoO2@Ni-felt after 100-hour single-cell test.

Table S7. Comparison of the HER performance of nPGM catalysts from literature at elevated temperatures and high alkaline concentrations (overpotentials at reported current densities; Tafel slopes where available)

Cathode catalyst	Preparation method	Conditions	Performance	Source
$Ni_{10}Mo/MoO_2$@Ni-foam-2h	Hydrothermal deposition + thermal reduction	30 wt. % KOH, 80 °C	-89 mV @ -1 A/cm²<br>52 mV/dec	This work
NiMo@stainless-steel	Electrodeposition	30 wt. % KOH, 80 °C	-98 mV @ -0.1 A/cm²<br>158.6 mV/dec	[3]
Ni-201 plate	Commercial	30 wt. % KOH, 91.4 °C	-457 mV @ -0.6 A/cm²<br>115 mV/dec	[4]
RANEY® nickel mesh	Commercial	30 wt. % KOH, 60 °C	-310 mV @ -0.4 A/cm²	[5]
Ni-Hcd[a]@stainless-steel	Electrodeposition	30 wt. % KOH, 80 °C	-285 mV @ -0.1 A/cm²<br>171.4 mV/dec	[6]
NiCo@stainless-steel	Electrodeposition	30 wt. % KOH, 80 °C	-339 mV @ -0.1 A/cm²<br>145 mV/dec	[6]

[a]Hcd = electrodeposited at high current density

Table S8. Comparison of the overall water splitting performance with catalysts from literature at elevated temperatures and high alkaline concentrations

**Cathode catalyst**	**Anode Catalyst**	**Conditions**	**Performance**	**Source**
$Ni_{10}Mo/MoO_2$@Ni-felt	NiFe-LDH@Ni-felt	30 wt. % KOH, 80 °C	1.58 V @ 0.67 A/cm² (initial),<br>+0.3 mV/h over 100 h @ 0.67 A/cm²	This work
RANEY®-type Ni-Mo	RANEY®-type Ni	24 wt. % KOH, 80 °C,<br>ion solvating membrane	1.8 V @ 1.7 A/cm²,<br>failed after 120 h	[7]
Mo-containing Raney Ni	Raney Ni/Co_3O_4	30 wt. % KOH, 80 °C	1.6 V @ 0.3 A/cm²,<br>no degradation over 15 000 h	[8]
NiMo@NM[a]	NiFe@NM[a]	6 M KOH, 70 °C	1.72 @ 0.4 A/cm² (initial),<br>+2.2 mV/h over 51 h	[9]
IrNi-$FeNi_3$@NF[b]	IrNi-$FeNi_3$@NF[b]	6 M KOH, 60 °C	1.85 V @ 0.5 A/cm² (initial),<br>+3 mV/h over 100 h	[10]
Pt/C	RP/SP[c]	6 M KOH, 60 °C, AEM	~1.7 V @ 0.2 A/cm² (initial),<br>~ +2.5 mV/h over 20 h	[11]

Ni/NiMo/rNS[d]	Ni/NiMo/rNS[d]	30 wt. % KOH, 70 °C	1.91 V @ 0.46 A/cm², stable over 120 h	[12]
NiFe@NF[b]	NiFe@NF[b]	6 M KOH, 80 °C	1.56 V @ 0.1 A/cm² (initial), +0.26 mV/h over 120 h	[13]

[a]NM = nickel mesh, [b]NF = nickel foam, [c]RP/SP = Ruddlesden-Popper/single perovskite, [d]rNS = roughened nickel sheet

REFERENCES

(1) Zhang, J.; Wang, T.; Liu, P.; Liao, Z.; Liu, S.; Zhuang, X.; Chen, M.; Zschech, E.; Feng, X. Efficient hydrogen production on MoNi4 electrocatalysts with fast water dissociation kinetics. *Nature Communications* **2017**, *8* (1), 15437. DOI: 10.1038/ncomms15437. Published Online: May. 17, 2017.

(2) Rauscher, T.; Müller, C. I.; Gabler, A.; Gimpel, T.; Köhring, M.; Kieback, B.; Schade, W.; Röntzsch, L. Femtosecond-laser structuring of Ni electrodes for highly active hydrogen evolution. *Electrochimica Acta* **2017**, *247*, 1130–1139. DOI: 10.1016/j.electacta.2017.07.074.

(3) González-Buch, C.; Herraiz-Cardona, I.; Ortega, E.; García-Antón, J.; Pérez-Herranz, V. Study of the catalytic activity of 3D macroporous Ni and NiMo cathodes for hydrogen production by alkaline water electrolysis. *J Appl Electrochem* **2016**, *46* (7), 791–803. DOI: 10.1007/s10800-016-0970-0.

(4) Demnitz, M.; Lamas, Y. M.; Garcia Barros, R. L.; Leeuw den Bouter, A. de; van der Schaaf, J.; Theodorus de Groot, M. Effect of iron addition to the electrolyte on alkaline water electrolysis performance. *iScience* **2024**, *27* (1), 108695. DOI: 10.1016/j.isci.2023.108695. Published Online: Dec. 10, 2023.

(5) Di, F.; Chen, C.; Shen, J.; Wei, Z.; Dong, W.; Peng, Y.; Fan, R.; Shen, M.; Olu, P.-Y. Dynamic and interconnected influence of dissolved iron on the performance of alkaline water electrolysis. *Chemical science* **2025**, *16* (22), 9913–9919. DOI: 10.1039/d5sc01380a. Published Online: Apr. 29, 2025.

(6) Herraiz-Cardona, I.; Ortega, E.; Antón, J. G.; Pérez-Herranz, V. Assessment of the roughness factor effect and the intrinsic catalytic activity for hydrogen evolution reaction on Ni-based electrodeposits. *International Journal of Hydrogen Energy* **2011**, *36* (16), 9428–9438. DOI: 10.1016/j.ijhydene.2011.05.047.

(7) Kraglund, M. R.; Carmo, M.; Schiller, G.; Ansar, S. A.; Aili, D.; Christensen, E.; Jensen, J. O. Ion-solvating membranes as a new approach towards high rate alkaline electrolyzers. *Energy Environ. Sci.* **2019**, *12* (11), 3313–3318. DOI: 10.1039/C9EE00832B.

(8) SCHILLER, G. High performance electrodes for an advanced intermittently operated 10-kW alkaline water electrolyzer. *International Journal of Hydrogen Energy* **1998**, *23* (9), 761–765. DOI: 10.1016/s0360-3199(97)00122-5.

(9) Zhu, L.; Fang, Q.-Y.; Liu, S.-T.; Li, B.; Li, F.; Guo, Z.-G.; Deng, N.; He, J.-B. Two closed-loop nickel-based catalysts for use in alkaline water electrolysis under industrial conditions. *J Solid State Electrochem* **2024**, *28* (10), 3915–3927. DOI: 10.1007/s10008-024-05996-2.

(10) Wang, Y.; Qian, G.; Xu, Q.; Zhang, H.; Shen, F.; Luo, L.; Yin, S. Industrially promising IrNi-FeNi3 hybrid nanosheets for overall water splitting catalysis at large current density. *Applied Catalysis B: Environmental* **2021**, *286*, 119881. DOI: 10.1016/j.apcatb.2021.119881.

(11) Tang, J.; Xu, X.; Tang, T.; Zhong, Y.; Shao, Z. Perovskite-Based Electrocatalysts for Cost-Effective Ultrahigh-Current-Density Water Splitting in Anion Exchange Membrane Electrolyzer Cell. *Small Methods* **2022**, *6* (11), e2201099. DOI: 10.1002/smtd.202201099. Published Online: Oct. 17, 2022.

(12) Chen, Y.; Wang, Y.; Yu, J.; Xiong, G.; Niu, H.; Li, Y.; Sun, D.; Zhang, X.; Liu, H.; Zhou, W. Underfocus Laser Induced Ni Nanoparticles Embedded Metallic MoN Microrods as Patterned Electrode for Efficient Overall Water Splitting. *Advanced science (Weinheim, Baden-Wurttemberg, Germany)* **2022**, *9* (10), e2105869. DOI: 10.1002/advs.202105869. Published Online: Feb. 3, 2022.

(13) Feng, L.; Zhou, J.; Xiao, J.; Chen, F.; Zhao, Z.; Liu, M.; Zhang, N.; Gao, F. Simple cathodic deposition of FeS/NiS-activated Ni/NiO heterojunctions for high-concentration

overall water splitting reactions. *International Journal of Hydrogen Energy* **2023**, *48* (77), 29852–29864. DOI: 10.1016/j.ijhydene.2023.04.077.
